# Highly active and stable OER electrocatalysts derived from Sr_2_MIrO_6_ for proton exchange membrane water electrolyzers

**DOI:** 10.1038/s41467-022-35631-5

**Published:** 2022-12-24

**Authors:** María Retuerto, Laura Pascual, Jorge Torrero, Mohamed Abdel Salam, Álvaro Tolosana-Moranchel, Diego Gianolio, Pilar Ferrer, Paula Kayser, Vincent Wilke, Svenja Stiber, Verónica Celorrio, Mohamed Mokthar, Daniel García Sanchez, Aldo Saul Gago, Kaspar Andreas Friedrich, Miguel Antonio Peña, José Antonio Alonso, Sergio Rojas

**Affiliations:** 1 Grupo de Energía y Química Sostenibles, Instituto de Catálisis y Petroleoquímica, CSIC. C/Marie Curie 2, 28049 Madrid, Spain; 2 Instituto de Catálisis y Petroleoquímica, CSIC. C/Marie Curie 2, 28049 Madrid, Spain; 3 Institute of Engineering Thermodynamics/Electrochemical Energy Technology, German Aerospace Center (DLR), Pfaffenwaldring 38-40, 70569 Stuttgart, Germany; 4 Chemistry Department, Faculty of Science, King Abdulaziz University, P. O Box 80200, Jeddah, 21589 Saudi Arabia; 5 Diamond Light Source, Harwell Science and Innovation Campus, Didcot, OX11 0DE UK; 6 Instituto de Ciencia de Materiales de Madrid, CSIC. C/Sor Juana Inés de la Cruz 3, 28049 Madrid, Spain

**Keywords:** Electrocatalysis, Electrocatalysis, Nanoscale materials

## Abstract

Proton exchange membrane water electrolysis is a promising technology to produce green hydrogen from renewables, as it can efficiently achieve high current densities. Lowering iridium amount in oxygen evolution reaction electrocatalysts is critical for achieving cost-effective production of green hydrogen. In this work, we develop catalysts from Ir double perovskites. Sr_2_CaIrO_6_ achieves 10 mA cm^−2^ at only 1.48 V. The surface of the perovskite reconstructs when immersed in an acidic electrolyte and during the first catalytic cycles, resulting in a stable surface conformed by short-range order edge-sharing IrO_6_ octahedra arranged in an open structure responsible for the high performance. A proton exchange membrane water electrolysis cell is developed with Sr_2_CaIrO_6_ as anode and low Ir loading (0.4 mg_Ir_ cm^−2^). The cell achieves 2.40 V at 6 A cm^−2^ (overload) and no loss in performance at a constant 2 A cm^−2^ (nominal load). Thus, reducing Ir use without compromising efficiency and lifetime.

## Abstract (web summary)

While water splitting offers a renewable means to produce H_2_ fuel, most electrolyzers rely on scarce elements to function. Here, authors study low-content Iridium catalysts derived from mixed oxides for proton exchange membrane water electrolysis anodes without compromising activity and durability.

## Introduction

Green hydrogen, *i*.*e*., the hydrogen produced through water electrolysis from renewable energy, has been identified as the key sustainable energy carrier for decarbonizing industry and transport sectors, including hard-to-abate subsectors. The demand for green hydrogen is therefore expected to increase drastically in the coming years from today’s approximately 1% to 12% by 2050^1^. Proton exchange membrane water electrolysis (PEMWE) is regarded as the ideal technology to transform renewable energy into hydrogen^2,3^. PEMWE technology presents several advantages, including a fast response to fluctuations in renewable energies; operation at high current densities; production of high purity H_2_ since the gas crossover rate is low; production of pressurized hydrogen during electrolysis reducing operational costs; and a compact design that is easy to stack and scale.

The electrolysis process is the splitting of H_2_O, into H_2_ and O_2_ using electricity. In a PEMWE, H_2_ is formed at the cathode (4H^+^ + 4e^−^ → 2H_2_), and O_2_ is produced at the anode (2H_2_O → O_2_ + 4H^+^ + 4e^−^)^4^. The latter reaction, *i*.*e*., the oxygen evolution reaction (OER), is the limiting process, requiring large amounts of electrocatalyst to take place at reasonable overpotentials^5^. Due to the very strongly oxidizing environment in the anode of a PEMWE (low pH, high oxygen concentration, high potential and the presence of water), OER catalysts are based on Ru- or Ir-oxides. Although Ru catalysts display high initial activity, they are unstable during the OER^6^. Ir oxide-based catalysts display high OER activity and stability, with Ir-black, IrO_2_, IrO_x_-Ir, and IrNiO_x_ being among the most active catalysts for the OER^7,8^. However, Ir is a scarce metal that is extracted as a minor byproduct of platinum, and its demand is growing (267,000 oz in 2021)^9^; therefore, it is extremely expensive ($6300 per oz in 2021), with both its price and production subjected to strong fluctuations, posing a risk for the deployment and scaling up of PEM electrolyzers. Therefore, a great deal of interest has been placed on reducing the content of Ir in OER electrocatalysts.

Ir mixed oxides display comparable or higher OER Ir mass-specific activities than Ir simple oxides^10–15^. However, Ir mixed oxides lack structural stability during the OER, mainly due to the high solubility of the non-noble elements in aqueous-acid solutions. In recent years, many efforts have been devoted to understanding this effect and to identifying the species formed during the OER, since the performance of Ir-mixed oxides is not the same between them so the reconstructions have to be different. Most works in the literature report the formation of simple iridium phases such as Ir-O-OH, Ir-OH, IrO_x_ and/or IrO_2_ with different levels of amorphization, although the exact nature of such phases remains unknown^12,16–20^.

In this work, we synthesized Ir double perovskites, Sr_2_CaIrO_6_, Sr_2_MgIrO_6_, and Sr_2_ZnIrO_6_ with the Ir atoms in a high oxidation state (Ir^6+/5+^). We monitored the stability of the catalysts during the OER using both in situ and ex situ approaches. We observed a reconstruction of the surface of the perovskites due to the rapid dissolution of alkaline cations, especially Ca, produced after immersion in the electrolyte and progresses during the first OER cycles under it stabilizes. Despite the removal of the alkaline cations, the skeleton of the original perovskite remains unaltered and the surface reconstructs into short-ordered corner and edge-sharing IrO_6_ octahedra in a very open structure responsible for the high OER activity of Sr_2_CaIrO_6_, which is among the highest reported in the literature, especially when it is tested in a PEM electrolyzer.

## Results and discussion

### Crystal structure of the double perovskites

Sr_2_MIrO_6_ (M = Ca, Zn, Mg) displays an A_2_BB´O_6_ double perovskite structure with P*2*_*1*_*/n* monoclinic symmetry (the Rietveld refinement of the crystal structures is shown in Figure S1, Supporting Information (SI)). Sr_2_MIrO_6_ shows a regular arrangement of alternating corner-sharing MO_6_ and IrO_6_ octahedra with Sr cations occupying the voids between the octahedra (inset of Fig. S1, SI). Sr_2_CaIrO_6_ presents 13(2)% disorder between the Ca and Ir along both B positions, while Sr_2_ZnIrO_6_ and Sr_2_MgIrO_6_ present slightly larger cationic disorder. In fact, the values of the interatomic distances in Sr_2_CaIrO_6_ (<Ir–O> = 1.937(5) Å and <Ca–O> = 2.248(5) Å) differ more between them than in the other two oxides (Table S2, SI), which explains the larger cationic ordering and indicates the stronger asymmetric character of the Ir-O-Ca bond and strong anion polarization. The values of the distances using XRD have to be taken carefully since oxygen does not have a strong x-ray scattering effect. However, these results are comparable to previous results reported by the group using neutron powder diffraction^21,22^, which is very reliable to differentiate between, for instance, Sr/Ca, Sr/Mg or oxygen positions and occupancy. The volume and cell parameters decrease in the order Ca^2+^> Zn^2+^> Mg^2+^, which is in line with the evolution of the ionic radii of M^2+^ cations.

The surface composition and oxidation state of the Ir surface atoms of Sr_2_MIrO_6_ were analyzed by XPS. A thorough discussion of the assignment of the peaks of the Ir 4*f* core-level region can be found in section S4, SI. The binding energy (BE) of the Ir 4*f*_7/2_ core level in Sr_2_CaIrO_6_ is centered at approximately 64.4 eV, shifting to lower BE of approximately 62.7 eV for Sr_2_MgIrO_6_ and Sr_2_ZnIrO_6_. This is due to the different contributions of the Ir^5+^/Ir^6+^ and Ir^4+^/Ir^3+^ components (Fig. S4, SI), with a higher contribution of Ir^5+^/Ir^6+^ in Ca perovskite in comparation with Mg and Zn perovskites. These values are in line with the BE values reported for Ir perovskites with Ir^5+^ and Ir^6+^, such as La_2_LiIrO_6_ and Ba_2_PrIrO_6_^12,23^, suggesting the presence of Ir^6+^ in Sr_2_CaIrO_6_ and Ir^5+^/Ir^6+^ in Sr_2_MgIrO_6_ and Sr_2_ZnIrO_6_. This assignment is in line with the oxidation state obtained by XAS for these perovskites^21,22^.

The crystalline domain sizes of the double perovskites were calculated by XRD, obtaining a value of approximately 30 nm for all samples (Figure S1, SI). However, the TEM micrographs show the presence of larger particles (approximately 300 nm (Fig. S2, SI)), indicating that such large particles are formed by the agglomeration of smaller crystallites (Table S3, SI).

### Electrochemical performance. OER activity and durability in RDE

**Fig. 1:**
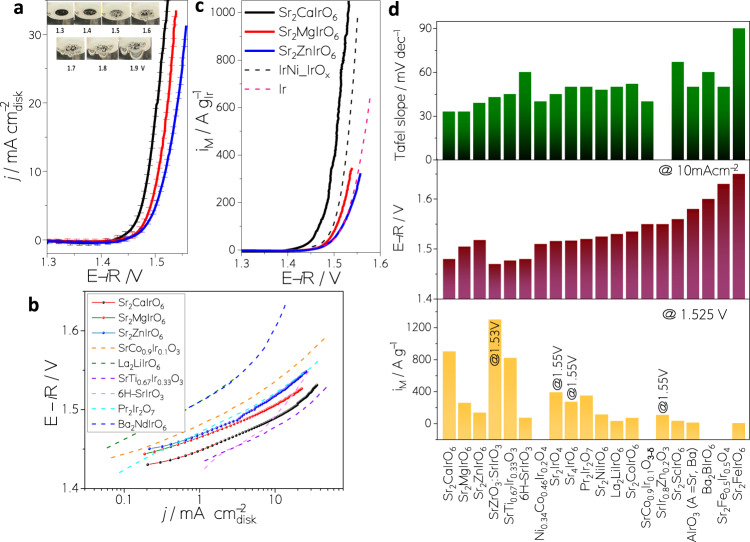
OER performance. **a** Current densities obtained for Sr_2_MIrO_6_ (M = Ca, Mg and Zn). Inset: Generation of oxygen in Sr_2_CaIrO_6_ in the RDE configuration at different potentials. **b** Tafel plots of the Sr_2_MIrO_6_ catalysts and SoA catalysts from refs. [12–14,19,23,59]. **c** Ir mass-specific OER activity for Sr_2_MIrO_6_ compared to catalysts of reference. ^7^ **d** Tafel slope (upper panel), potential at 10 mA cm^−2^ (middle panel) and Ir mass-specific activity at 1.525 V (bottom panel) of the state-of-the-art OER Ir-mixed oxide catalysts reported in the literature (appropriate references are given in Table S1).

Figure 1a depicts the *i*R-corrected polarization curves for the catalysts normalized to the geometric area of the electrode. The OER activity follows the order Sr_2_CaIrO_6_ > Sr_2_MgIrO_6_ > Sr_2_ZnIrO_6_. A metric usually reported to benchmark the OER activity is the potential at a current density of 10 mA cm^−2^ ^24^. Sr_2_CaIrO_6_ reaches 1.48 V to achieve this current density and Sr_2_MgIrO_6_ and Sr_2_ZnIrO_6_ 1.49 V and 1.51 V, respectively. Note that the formation of bubbles over Sr_2_CaIrO_6_ is visible at 1.4 V (Inset Fig. 1a). The potential to achieve 10 mA cm^−2^ with Sr_2_CaIrO_6_ is similar to the ones reported for the best performing Ir-mixed oxides in the literature, namely, SrTi_0.67_Ir_0.33_O_3,_ 6H-SrIrO_3_ and SrZrO_3_:SrIrO_3_ (Zr:Ir 1:2)^13,14,25^, see Fig. 1d and Table S1 in the SI. Tafel slopes of approximately 33–38 mV dec^−1^ (Fig. 1b) were obtained for Sr_2_MIrO_6_ (M = Ca, Mg, Zn). The low Tafel slopes indicate a fast kinetics of the catalysts, superior to most Ir mixed oxides (Fig. 1d). In order to assess the specific catalytic activities, the currents have been normalized to the mass-specific surface areas (*A*_*S*_), see Section S4.1. The results show that the specific activity of Sr_2_CaIrO_6_ is higher than that of Sr_2_MgIrO_6_ and Sr_2_ZnIrO_6_ (Fig. S7, SI).

Concerning the Ir mass-specific activities, Sr_2_CaIrO_6_ reaches 900 A g^−1^_Ir_ at 1.525 V, with Sr_2_MgIrO_6_ and Sr_2_ZnIrO_6_ reaching Ir mass-specific activity of 260 and 139 A g^−1^_Ir_, respectively (Fig. 1c). Figure 1d and Table S1 compares these values with the literature, being Sr_2_CaIrO_6_ only surpassed by SrZrO_3_:SrIrO_3_ among Ir mixed oxides^25^. Finally, we obtained a turnover frequency (TOF) for Sr_2_CaIrO_6_ at 1.5 V of 0.71 s^−1^ (see Section S6.5 in the SI for details concerning TOF calculation). This value compares well with values reported for IrO_2_ particles^8^.

To evaluate the durability in RDE, Fig. S8a in the SI depicts the initial OER activity of the three perovskites and their activity after 100 cycles. Since the activity remained stable, the most active catalyst, Sr_2_CaIrO_6_, was subjected to a longer stability test of 5000 OER cycles, recording a loss of 15% of its initial activity. Chronoamperometry tests were performed for 1 h at the potentials needed to reach a current density of 10 mA cm^−2^ (not *i*R corrected) and the activities remain stable during the tests (Fig. S8b, SI).

The origin of the high OER activity of the Sr_2_MIrO_6_ catalysts has been previously ascribed to the presence of Ir atoms in the high oxidation state, namely, 6+ and 5+, which are reported to display high OER activity^12,26^. However, considering Ir oxidation state as the only descriptor of the OER activity should be taken cautiously since Ir^5+/6+^ cations only stabilize in certain mixed oxides that are unstable during OER under acidic conditions and result in the formation of Ir^3+/4+^ phases. Recent reports suggest that the long-term OER activity of iridium mixed oxides accounts for the simple iridium oxide phases formed during the OER^16–20^, especially short-range order phases, thus questioning the relevance of the oxidation state of Ir in the mixed oxide^27^. However, this view fails to explain why the OER activities of iridium mixed oxides depend on their initial composition and structure. In this work, for instance, Sr_2_CaIrO_6_ records higher activity than Zn and Mg catalysts. In view of this, we carefully monitored the evolution of Sr_2_CaIrO_6_ (the most active catalyst reported in this work) during different stages of the reaction with the aim of unveiling the evolution and nature of the iridium oxide phases formed during the OER.

### Evolution of Sr_2_CaIrO_6_ after immersion in 0.1 M HClO_4_ electrolyte

**Fig. 2:**
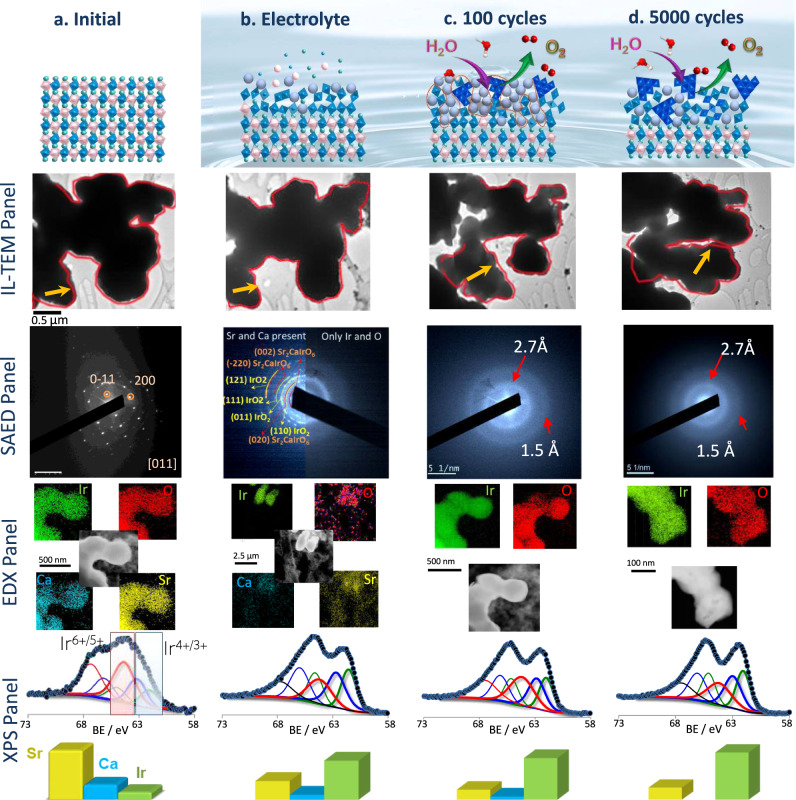
Evolution of Sr_2_CaIrO_6_ in the electrolyte and during the OER. **a** Fresh Sr_2_CaIrO_6_, (**b**) immersed in the electrolyte (Sr_2_CaIrO_6_-Elec), (**c**) after 100 OER cycles (Sr_2_CaIrO_6_-100) and (**d**) after 5000 OER cycles (Sr_2_CaIrO_6_-5000). Upper panel: Schematic view of the surface reconstruction. Central Panels: IL-TEM micrographs of the same region, SAED and EDX. Bottom Panel: XPS spectra showing the evolution of the Ir 4*f* region and the relative surface atomic composition of Sr, Ca, and Ir.

A reconstruction of the catalyst surface commences after immersion of the perovskite in the acidic electrolyte. Figure 2 shows the selected identical location-TEM (IL-TEM panel) images of Sr_2_CaIrO_6_ obtained before (Fig. 2a, IL-TEM panel) and after 5 min of immersion in 0.1 M HClO_4_ (Fig. 2b, IL-TEM panel), named Sr_2_CaIrO_6_-Elec. Before discussing the results, it is important to note that TEM analysis of the very large particles was not possible due to the intrinsic limitations of the technique. The shape of the particles remains stable, but a slight decrease in the size of the particles (approximately 5%) can be observed. The elemental EDX mappings of Sr_2_CaIrO_6_ and Sr_2_CaIrO_6_-Elec are shown in Fig. 2a, b (EDX panel). As expected, fresh Sr_2_CaIrO_6_ displays a homogeneous and stoichiometric distribution of Sr, Ca, Ir and O across the particles. On the other hand, the EDX mappings of Sr_2_CaIrO_6_-Elec (Fig. 2b and Fig. S10, SI) reveal a heterogeneous distribution of the elements, showing regions with Sr, Ca, Ir and O (with a partial loss of Ca and Sr), along with regions where only Ir and O can be found. The SAED analysis of the regions containing Sr, Ca, Ir and O shows several diffraction rings, corresponding to Sr_2_CaIrO_6_ and IrO_2_ (Fig. 2b left, SAED panel). On the other hand, the SAED of the particles containing only Ir and O shows two broad rings (2.7 and 1.5 Å) ascribed to amorphous IrOOH (Fig. 2b right, SAED panel)^28,29^.

The dissolution of Sr and Ca was confirmed by analysis of the composition of the electrolyte by ICP-OES (Table S4, SI). After 10 min of immersion in 0.1 M HClO_4_ approximately 50% of Ca, along with a small fraction of Sr, dissolves in the electrolyte.

The surface composition of Sr_2_CaIrO_6_-Elec was analyzed by XPS (Fig. 2b, XPS panel and Fig. S11, SI). The relative surface concentration of Ca and Sr decreases, confirming their dissolution. The Ir 4*f* core-level spectrum of Sr_2_CaIrO_6_-Elec shifts to a lower binding energy than that of the fresh sample (Figure S12, SI). The deconvolution of the spectrum reveals that the shifting accounts for the increasing intensity of the peaks ascribed to Ir^3+^ and Ir^4+^ species, most likely IrOOH and IrO_2_, which become the main Ir species on the surface of Sr_2_CaIrO_6_-Elec. Nonetheless, Ir^5+^/Ir^6+^ species are still observed, indicating that either the extension of the surface reconstruction is limited to a few layers (note that the XPS signal carries information from several layers of the catalyst, a depth of approximately 2–3 nm) and/or particles of the original perovskite that resist to the electrolyte. The analysis of the O 1 *s* core-level spectra is shown in Fig. S13, SI. Similar results were obtained for Sr_2_ZnIrO_6_ (see Table S4, Figs. S11–S13, SI); however, the extend of the dissolution of Zn catalyst is less significant than that observed for the Ca catalyst.

The presence of the perovskite phase in Sr_2_CaIrO_6_-Elec was further confirmed by XRD (Fig. S14, SI). The absence of diffraction lines for the Ir^4+^/Ir^3+^ phases reveals that the IrOOH and IrO_2_ phases in the surface are very amorphous, lacking long-range order. The evolution of the structure during immersion in the electrolyte was further monitored with in situ XAS. Figure S15 in the SI shows the Ir L_3_-edge spectra for Sr_2_CaIrO_6_ and Sr_2_CaIrO_6_-Elec. The small change in the intensity of the edge indicates that immersion in the electrolyte leads to a change in Ir coordination. The low intensity of the change indicates that only a small fraction of the material is subjected to this change. There is no clear shift in the peak, so the overall oxidation state of the Ir atoms in Sr_2_CaIrO_6_ remain stable.

In summary, the reconstruction of Sr_2_CaIrO_6_ commences during immersion in the electrolyte. The dissolution of the Ca and Sr cations leads to the formation of several surface layers of amorphous Ir-O_x_-H_y_ phases in which Ir is more reduced than in the original perovskite. The extent of this transformation is limited to the surface, with most of the perovskite particles remaining unaltered during immersion.

### Evolution of Sr_2_CaIrO_6_ after 100 OER cycles

The stability of Sr_2_CaIrO_6_ was studied by analyzing the composition and structure of the catalyst recovered after 100 OER cycles (Sr_2_CaIrO_6_-100) at 10 mV s^−1^ between 1.2 and 1.7 V *vs*. RHE. The XPS analysis of Sr_2_CaIrO_6_-100 reveals that the loss of Ca and Sr cations from the catalyst surface is more severe, with most of the Ir atoms in Ir^3+^/Ir^4+^ oxidation states, although Ir^5+/6+^ species are also observed (Fig. 2c, XPS panel and Figs. S11 and S12, SI). Note that the XRD of Sr_2_CaIrO_6_-100 reveals that the bulk crystalline structure is still the perovskite (Fig. S14d, SI) and that diffraction lines for IrO_2_ or IrOOH are not observed. However, it can be noticed a slight decrease of the crystalline domain size of the perovskites with cycling (Table S6).

**Fig. 3:**
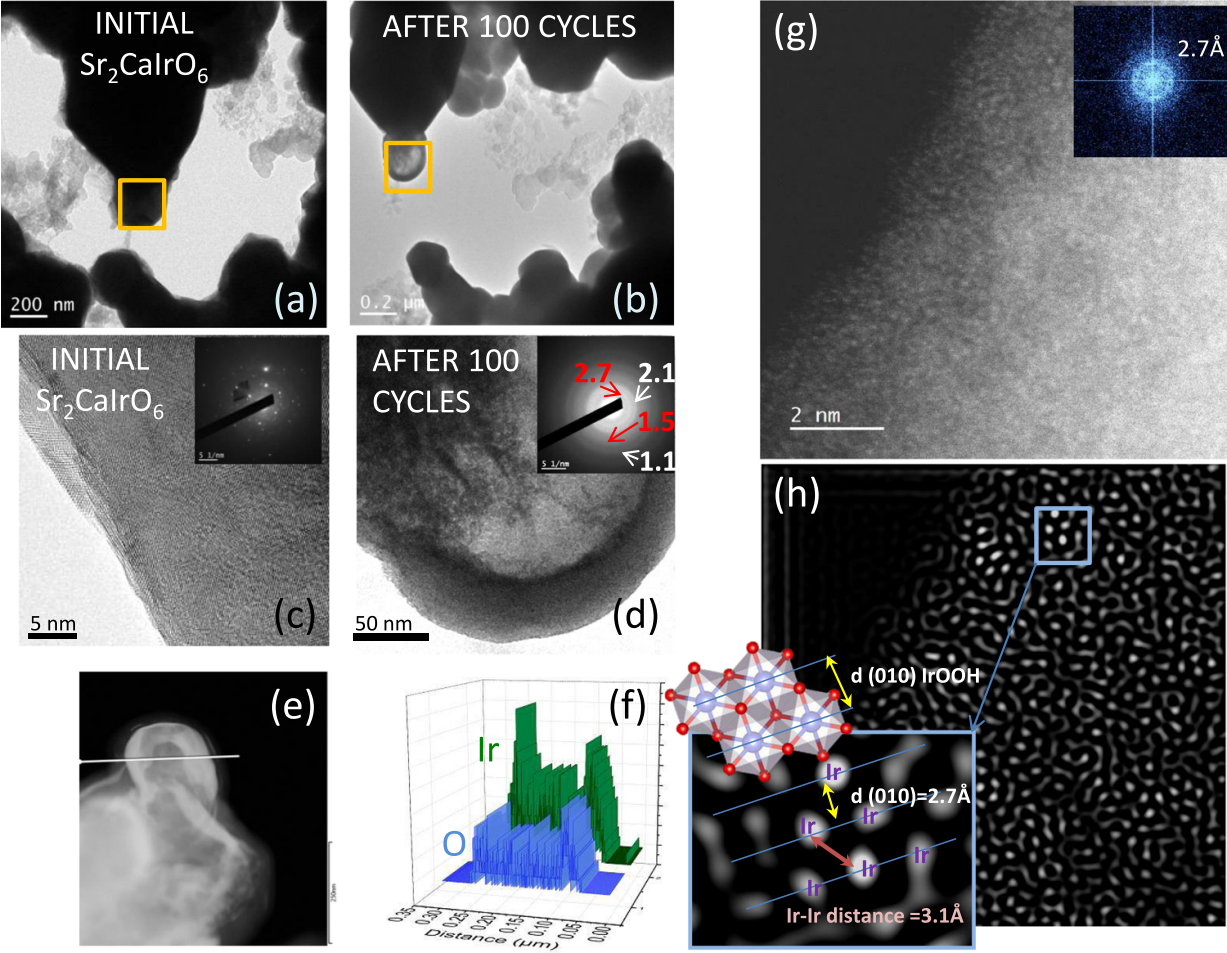
Morphology and structure of the hollow Ir-O particles. IL-TEM of a region of Sr_2_CaIrO_6_ (**a**) before and (**b**) after 100 OER cycles. (**c**) HRTEM of the initial catalyst. **d**–**f** TEM, STEM and EDX images of the hollow regions composed of Ir and O. Inset of Fig.d: SAED pattern. Note that diffraction rings at 2.1 and 1.1 Å appear after long-term exposure due to Ir^0^ species formed by the reduction of Ir^n+^ phases under the electron beam^31^. **g** Aberration-corrected STEM-HAADF image and (h) the corresponding filtered image with a magnification of a short-range ordered region between Ir cations of the catalyst after 100 cycles.

Figure 2c (IL-TEM panel) shows that the general shape of the particles after 100 cycles remains unaltered. However, a closer inspection of the images reveals that the particles evolved to a hollow-open structure, indicating that the dissolution of Ca and Sr does not result in the collapse of the perovskite structure, forming a dense material; instead, the skeleton of the perovskite remains stable (Fig. 3). EDX analysis of these hollow regions (Fig. 3f) reveals that they only contain Ir and O, and their SAED shows the broad diffraction rings ascribed to IrOOH (Inset Fig. 3d). In fact, the formation of these open structures is in line with the larger mass-specific surface areas obtained from the ECSA after immersion of the catalysts in the electrolyte compared to the fresh catalysts (see further discussion in Table S3 and Section S4.2).

Note that the formation of this kind of open structures is less evident for Mg and Zn perovskites (Fig. S18, SI), possibly because the dissolution of Mg and Zn cations is slower than Ca. Also, this kind of reconstruction was not observed for similar Ir-mixed oxides. For instance, SrCo_0.9_Ir_0.1_O_3-δ_, Sr_2_IrO_4_ and R_2_Ir_2_O_7_ reconstruct in corner-shared and under-coordinated IrO_6_ octahedrons, responsible for their high activities^16,17,19^; SrIr_0.8_Zn_0.2_O_3_ perovskite experiences an OER-induced metal to insulator transition that reduces its OER activity after 800 cycles^20^; in other oxides, such as Sr_2_CoIrO_6_, there is a deposition of an IrO_x_ layer on the surface^26,30^; La_2_LiIrO_6_ evolves into IrO_2_ particles at the surface^12^; and 9R-BaIrO_3_ shows the initial formation of IrO_*x*_ nanoparticles, which evolve into amorphous Ir^4+^O_*x*_H_*y*_/IrO_6_ octahedrons and then to amorphous Ir^5+^O_x_/IrO_6_ octahedrons on the surface^18^. This observation suggests that the nature of the cation at the B sites, rather than Sr at the A sites, contributes to the stability and nature of the phases formed during the reconstruction. The larger size, different nature, and the asymmetric character of the Ir-O-Ca bonds, make Ca^2+^ cations less likely to be accommodated at the B sites; being prone to be removed faster than the other cations during the process. The rapid dissolution of Ca leads to voids that are not occupied by vicinal Ir cations, probably because they are rapidly filled by hydroxonium (H_3_O^+^) ions^16,31–33^, thereby stabilizing the hollow structure of nanosized (short-range order) clusters of IrO_6_ octahedra.

The local morphology of the hollow regions was further studied by aberration-corrected STEM-HAADF. These regions display a short-range order of a few angstroms, as shown in the Fourier fast transform (FFT) and in the filtered image (Fig. 3g, h). These small domains are formed by groups of Ir cations with interplanar distances of 2.7 Å similar to those of d(101) of IrOOH, in which IrO_6_ octahedra share edges (see inset of Fig. 3h). Previous reports have discussed the correlation between the OER activity and the structure (connection) between the IrO_6_ octahedra, with edge-sharing^29,33–36^ and face-sharing^13,34^ octahedra displaying the highest OER activity. The aberration-corrected analyses, and the in situ XAS experiments (see below) show that edge-sharing IrO_6_ octahedra are the main phase in the hollow regions of the reconstructed catalyst. As discussed elsewhere, see references above, edge-sharing IrO_6_ octahedra with shorter Ir-Ir distances display high OER performance.

### Evolution of Sr_2_CaIrO_6_ after 2000-5000 OER cycles

**Fig. 4:**
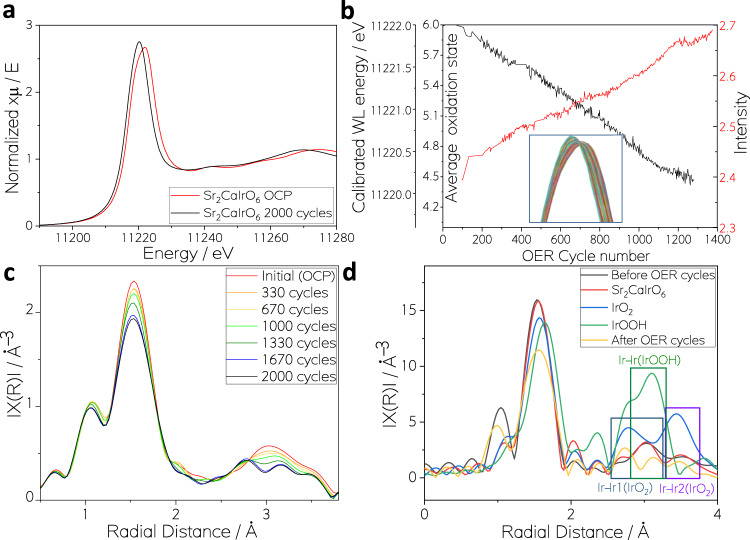
In situ monitoring of the catalyst evolution during the OER. **a** XANES signals of the Ir L_3_-edge of Sr_2_CaIrO_6_-Elec, Sr_2_CaIrO_6_−2000, and IrO_2_ standard. **b** Evolution of the white line position, related to the evolution of the oxidation state (black) and the intensity (red) of the XANES signal around 11220 eV during cycling. Inset: Zoom on white line position and intensity changes of the XANES signal observed in the whole series of Sr_2_CaIrO_6_ spectra collected during cycling. **c** Evolution of the FT-EXAFS region during OER cycling. **d** FT-EXAFS of Sr_2_CaIrO_6_-Elec, Sr_2_CaIrO_6_−2000 together with the simulated data of Sr_2_CaIrO_6_, IrO_2_ and IrOOH.

The evolution of Sr_2_CaIrO_6_ during 2000 OER cycles (Sr_2_CaIrO_6_-2000) was monitored by in situ XAS. The Ir L_3_-edge XANES signal of Sr_2_CaIrO_6_-Elec and Sr_2_CaIrO_6_-2000 are shown in Fig. 4a together with IrO_2_ standard. Figure 4b shows a shift of the white line position towards lower energies and an increment of the white line intensity at 11220 eV on spectra measured during the 2000 cycles, showing the correlation of this trend with a gradual change of Ir oxidation state and coordination geometry during the OER, excluding a quick phase transition. The energy shift from 11222.0 eV to 11220.1 eV indicates a partial reduction of Ir from 6+ to 4 + , consistent with the gradual formation of IrOOH and/or IrO_2_ species on the surface of Sr_2_CaIrO_6_. In fact, IrO_2_ white line position has been measured at 11219.6 eV.

The evolution of the Fourier transform (FT) from the Ir L_3_-edge extended X-ray absorption fine structure (EXAFS) signals during cycling is depicted in Fig. 4c. First, the intensity of the first coordination shell progressively decreases with increased cycling. This is consistent with the higher intensity of the first coordination shell of Sr_2_CaIrO_6_ than that of IrOOH. However, more evident differences are observed in the second coordination shell region, between 2.5 and 3 Å. To evaluate the changes in this region, the EXAFS spectra of Sr_2_CaIrO_6_, IrO_2_ and IrOOH have been simulated from reference data and compared with Sr_2_CaIrO_6_-Elec and Sr_2_CaIrO_6_-2000 (Fig. 4d). The spectrum of Sr_2_CaIrO_6_-Elec was fitted starting from a crystallographic structure of the perovskite. The fit resulted in an excellent match between experimental and simulated data as it can be appreciated in Fig. S16 and Table S7. Due to the complexity of the material, the presence of mixed phases, and the interference of contributions from a high number of scattering paths, a proper fit on the spectrum of Sr_2_CaIrO_6_-2000 could not be performed. Nevertheless, it is worth noticing that the signals of Sr_2_CaIrO_6_-2000 show an evolution across the whole radial distance range, but more evident in the second coordination shell region, where contributions from Ir-Ir paths for IrO_2_ and IrOOH (see Fig. 4d and Figure S17)^8,27,37^. This observation is compatible with the formation of IrO_2_ and IrOOH phases, confirming the results obtained by TEM and XPS.

Finally, Sr_2_CaIrO_6_ recovered after 5000 OER cycles (Sr_2_CaIrO_6_-5000) was analyzed. As shown in Fig. 2d (IL-TEM panel) the particles appear more agglomerated. The observation of the open structures is more frequent than after 100 cycles (Fig. S19, SI). The SAED pattern of the hollow regions displays halos at 2.7 and 1.5 Å (Fig. 2d) more diffuse than those obtained for Sr_2_CaIrO_6_-100 and Sr_2_CaIrO_6_-Elec, which suggests the continuous loss of local ordering. The agglomeration of the particles and/or the loss of local ordering can explain the 15% loss in activity observed after 5000 cycles (Fig. S8a, SI). The XPS and ICP-analysis of the electrolyte of Sr_2_CaIrO_6_-5000 are similar to that of Sr_2_CaIrO_6_-100, indicating that after 100 cycles the changes during OER are into a more amorphous morphology, but there is no more cations dissolution.

### Performance and stability of a Sr_2_CaIrO_6_ anode during PEMWE

**Fig. 5:**
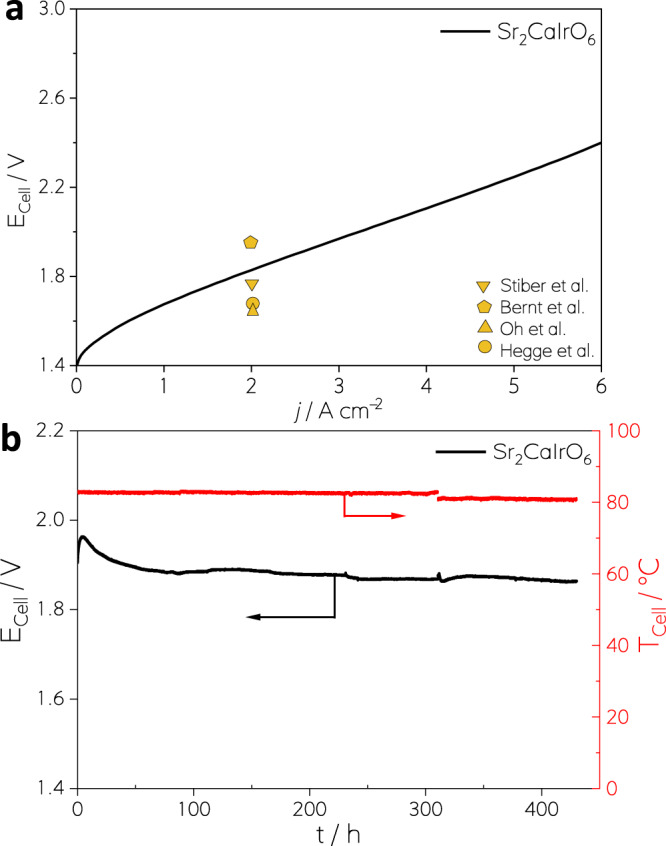
Assessment of OER performance during PEMWE. PEMWE measurements with the Sr_2_CaIrO_6_ anode (0.4 mg_Ir_ cm^−2^) and Pt/C (commercial) cathode (0.4 mg_Pt_ cm^−2^): (a) Cell potential (E_cell_) with respect to the current density (*j*) recorded galvanostatically up to 6 A cm^−2^ (overload) according to the JRC EU-harmonized procedure^58^; a dwell and consecutive recording period of 10 s was used for each current step. Reference performance values reached by Stiber et al.^43^ Oh et al.^44^, Bernt et al.^41^ and Hegge et al.^45^, using Nafion N212 based PEMWE CCMs are included. Note that^41,43,44^ use higher Ir loadings. **b** E_cell_ and cell temperature (*T*_cell_) evolution over time (*t*) (450 h) at a constant 2 A cm^−2^ (nominal load). The measurements were carried out at 80 °C and ambient pressure.

Since Sr_2_CaIrO_6_ recorded the highest OER activity in RDE among the studied oxides, it was chosen to be tested in the electrolysis cell. Catalyst-coated membranes (CCMs) employing Sr_2_CaIrO_6_ as the anode catalyst (1 mg_cat_cm^−2^ or 0.4 mg_Ir_ cm^−2^) were produced by spray coating, and then they were tested in a 4 cm^2^ active area PEMWE single cell. A polarization curve recorded up to 6 A cm^−2^ at 80 °C and ambient pressure is shown in Fig. 5a. The curve reveals a peak performance of 6 A cm^−2^ at 2.40 V and shows a linear slope, indicating the absence of significant mass-transport limitations or rapid degradation processes. This demonstrates that the high OER performance obtained in the RDE configuration is also attained in the electrolytic cell. With a cell potential of 1.81 V at the nominal current density of 2 A cm^−2^, the performance is comparable to the most recent reports of PEMWE^38–41^ with an Ir loading comparable or even lower than those used in commercial CCMs^42^. As shown in Fig. 5a, the performance of our cell is analogous to the best PEMWEs with the same MEA, i.e., Nafion 212, and operating conditions; 80 °C and ambient pressure^41,43–45^. Most of these studies were conducted using a higher Ir loading (1–2 mg_Ir_ cm^−2^) than our PEMWE (0.4 mg_Ir_ cm^−2^). Only recent works by Hegge et al.^45^ and Bernt et al.^46^ and Möckl et al.^47^ reported PEMWE with lower Ir loadings, also resulting in high performant CCMs. Given that none of the reports in the literature use 0.4 mg_Ir_ cm^−2^, an in-house reference CCM with commercial Ir_black_ (0.4 mg_Ir_ cm^−2^) has been produced using the coating media recipe reported by M. Bernt et al.^41^ The polarization curves, high-frequency resistances (HFR) and Tafel plots obtained are presented in Fig. S20. As deduced from the results presented in S13, the high cell performance can be mostly attributed to the enhanced electrochemical properties of the Sr_2_CaIrO_6_ electrode.

Last, to evaluate catalyst stability, a Sr_2_CaIrO_6_ anode cell was tested at a constant nominal load of 2 A cm^−2^. The recorded cell potential and cell temperature are shown in Fig. 5b. The cell shows an activation period during the first 50 h, but afterward, E_cell_ remains practically constant for 450 h, confirming that the electrocatalytic active phase derived from Sr_2_CaIrO_6_ is stable under PEMWE conditions, which was also shown in the RDE measurements.

In this work, we show that Sr_2_MIrO_6_ (Ca, Mg, Zn) perovskites, especially Sr_2_CaIrO_6_, generate OER electrocatalysts which are among the most active ones reported in the literature, as shown by the RDE and PEMWE test results. In order to understand the origin of such high activity, the evolution of Sr_2_CaIrO_6_ during the OER was thoroughly studied by ex situ and in situ techniques. During immersion in the electrolyte, and during the first OER cycles, the surface of the perovskite suffers severe reconstruction that is triggered by the dissolution of Ca and Sr cations into the electrolyte. This dissolution results in the formation of an outer layer of Ir-rich phases with Ir atoms in the 3^+^/4^+^ oxidation state. The fast removal of alkaline cations from the structure of the perovskite results in voids that are filled by H_3_O^+^ molecules, thus stabilizing the formation of the short-range ordered regions of edge-sharing IrO_6_ octahedra. The formation of such regions conforms an open structure at the surface of the perovskite which is the responsible for the very high OER activity and durability of the catalyst. The high OER activity and durability of the catalyst obtained from Sr_2_CaIrO_6_ allows for a significant reduction of the use of Ir down to 0.4 mg_Ir_ cm^−2^ at the anode of PEMWE. As a result, the bottleneck that represents the use of a scare material such as Ir for the scaling up and manufacturing PEMWEs can be avoided, making this technology the most suitable for the production of green hydrogen.

## Methods

### Synthesis of double perovskites

Sr_2_CaIrO_6_, Sr_2_MgIrO_6_ and Sr_2_ZnIrO_6_ were synthesized by sol-gel method following refs. [21,22] Sr(NO_3_)_2_, CaCO_3_, MgO, ZnO and IrO_2_ were dissolved in a solution of citric acid and HNO_3_ under strong magnetic stirring (note that IrO_2_ remained dispersed). The suspension was slowly evaporated at 100 °C leading to an organic resin in which the cations are homogeneously distributed. After complete evaporation of the solvents, the resulting resins were dried at 140 °C under static air and then heated at 600 °C under static air for 12 h in order to decompose the organic materials and eliminate the nitrates, obtaining reactive precursors. Then the precursors were then treated at 800 °C/12 in O_2_ flux. Subsequently, the compounds were heated under high oxygen pressure (200 bar) at 900 °C/12 h to obtain Ir^6+^ perovskite oxides.

### Physicochemical characterization

Phase identification and crystallite size were determined by x-ray powder diffraction (XRD). XRD patterns were collected on a X’PertProPAN analytical diffractometer using CuK_α_ radiation (λ = 1.5418 Å, 45 kV, 40 mA). The refinement of the crystal structures was performed using the Rietveld method and the Fullprof crystallographic program^48,49^.

Transmission electron microscopy (TEM), high-resolution electron microscopy (HRTEM), scanning transmission electron microscopy (STEM) and x-ray energy dispersive spectra (EDS) were recorded in a JEOL 2100 field emission gun transmission electron microscope operating at 200 kV and equipped with an EDX spectrometer Oxford INCA Energy 2000 system. The specimen was prepared by depositing aliquots of the desired sample onto a Cu grid supporting a lacey carbon film. Identical-Locations TEM (IL-TEM) was used to analyze the evolution of the catalysts at different stages during the OER^50^. To acquire the SAED patterns, low-dose illuminations conditions were used to avoid sample decomposition or other known effects such as the reduction of IrO_x_ to metallic Ir.

X-ray photoelectron spectra (XPS) were recorded with a VG Escalab200R electron spectrometer equipped with a Mg-Kα (hυ = 1253.6 eV) X-ray source. Powdered samples of the electrocatalysts were dispersed in a Nafion-free ink and deposited on a carbon double-sided adhesive tape supported on a stainless-steel holder. The same holder-supported catalyst was used in different electrochemical treatments: fresh catalyst, immersed in the electrolyte during 10 min, performing 5, 100, and 5000 cycles of OER between 1.2 and 1.7 V vs. RHE at 50 mVs^−1^. After every treatment, the sample was washed with water, dried at room temperature, and outgassed under a residual pressure of 10^−6^ mbar for 1 h in the XPS pre-chamber. Then, the samples were transferred into the analysis chamber and analysis begun when the residual pressure reached 10^−8^ mbar. The C1*s* peak due to the carbon double-sided adhesive tape and carbon black from the ink was set at 284.6 eV and used as reference. Peak intensities were estimated by calculating the integral of each peak after subtraction of a Shirley-shaped background and fitting the experimental peaks to a combination of Lorentzian and Gaussian curves. This fitting was based on bibliographic results of perovskite and related compounds; three different components were considered for the Ir 4*f*_7/2_ core level: Ir^0^/Ir^3+^, restricted to the range 61.2–62.2 eV, Ir^4+^, restricted to the range 62.2–63.2 eV, and Ir^5+^/Ir^6+^, restricted to the rage 63.2.0–64.4 eV (see S7, SI). Besides, a ratio of 3:4 was imposed for the area of the 4*f*_5/2_:4*f*_7/2_ spin-orbit doublets, which should be of the same FWHM for the same Ir species, and the constrained ranges for the 4*f*_5/2_ components were shifted by 3 eV. Finally, the FWHM was maintained under 2.5 eV for all the components. Surface composition was determined using the integrated peak areas and the corresponding Wagner sensitivity factors^51^.

X-ray Absorption Spectroscopy (XAS) measurements were performed at room temperature at Diamond Light Source (UK) on the B18 beamline^52^. Data were collected at Ir L_3_-edge (*E*  =  11215 eV) using a double crystal Si111 monochromator and Cr-coated mirrors. The measurements were performed in transmission mode using as detector 3 ion chambers with a gas mixture of Ar and He (80 mbar, 240 mbar, 240 mbar of Ar resulting in absorption of ca 15%, 50%, 50% respectively). For in situ XAS measurement, the samples were loaded into a custom-made electrochemical cell, available on B18 beamline and with a design based on the cell developed by Sardar et al.^53^ Electrochemical measurement were collected with an IVIUM potentiostat, using Au wire as counter electrode and Ag/AgCl as reference electrode. XAS data were collected in the energy range from 11015 to 11914 eV with a continuous QEXAFS acquisition mode and a constant energy step of 0.3 eV. The duration of a single scan was ca. 3 min. The scans were collected continuously while subjecting the sample to continuous cyclic voltammetry scans between 1.2 and 1.7 V_RHE_. XAS data treatment (including normalization, extraction of χ(k) and Fourier Transform) was performed with Athena software from Demeter package^54^. For the analysis of trends on the whole series of data a custom python script was used to monitor position and intensity of the normalized spectra whiteline. EXAFS signals for three reference structures were simulated by calculating the main scattering paths and their contributions with FEFF6 code^55^. The sum of most relevant paths was then calculated within Artemis software, using ΔE_0_ and Δr fixed to zero, S_0_^2^ fixed to 1 and Debye-Waller factors starting from 0.003 and increasing as a function of the path length R_eff_.

### Electrochemical characterization

An Autolab PGstat 302N potentiostat/galvanostat was used to test the electrochemical performance of the oxides. The measurements were performed using a standard three-compartment glass cell and a rotating disk electrode (RDE) (Pine Research Instruments). A graphite bar was used as the counter electrode. An Ag/AgCl (3M) electrode was used as the reference electrode. The oxides were deposited as an ink on a glassy carbon working electrode. The ink was prepared by mixing the oxides with carbon black (Vulcan-XC-72R) to improve the electrical conductivity. The mixture was dispersed in tetrahydrofuran (THF) and 5% Nafion and sonicated with an Ultrasonic Processor UP50H (Hielscher). The composition of the ink was 5 mg_oxide_, 1 mg_vulcan_, 0.03 mL_Nafion_ and 0.97 mL_THF_. 10 μL of ink were dropped onto the electrode of 0.196 cm^2^ of area, with a catalyst loading of 0.25 mg_oxide_ cm^−2^. Since pure perovskite phases were obtained, the Ir content in Sr_2_CaIrO_6_, Sr_2_MgIrO_6_ and Sr_2_ZnIrO_6_ is 38.2 wt.%, 39.4 wt.% and 36.3 wt.%, respectively. Therefore, the Ir loadings are 0.096, 0.099 and 0,090 mg_Ir_ cm^−2^ for Sr_2_CaIrO_6_, Sr_2_MgIrO_6_ and Sr_2_ZnIrO_6_, respectively.

The OER was initially assessed by recording cyclic voltammograms between 1.1 and 1.7 V_RHE_ at 10 mVs^−1^ and a rotation rate of 1600 rpm. The measurements were performed in an O_2_ saturated 0.1 M HClO_4_ electrolyte to assure the O_2_/H_2_O equilibrium at 1.23 V. The OER kinetic curves were capacitance-corrected by using the average of the anodic and cathodic curves and *i*R-corrected by using the formula *E*-*iR*_corrected_ = *E*_applied_ – *iR*. In this formula *i* is the current and *R* is the ohmic electrolyte resistance (R ~29 Ω) as obtained from Electrical Impedance Spectroscopy (EIS) at open circuit voltage. RDE durability tests were performed by recording 5000 consecutive cycles between 1.2 and 1.7 V_RHE_ at 10 mVs^−1^, or by recording a chronoamperometric program fixing the current density at 10 mA cm^−2^ and monitoring the evolution of the potential during 1 h. For the preparation of the cycled catalysts that are used for *post-mortem* characterization (XRD, XPS, etc) a large quantity of the catalysts mixed with vulcan were deposited on top of a carbon planchet. Then the catalysts were scraped off the planchet.

### CCM preparation and PEMWE measurements

The catalysts coated membranes (CCMs) were prepared by the wet spraying technique using a vacuum heating table (Fuel Cell Store) to hold the Nafion 212 PEM substrate in place and heat it to 100 °C during deposition. The distance between spraying nozzle and substrate was kept at. 6 cm, and the ink deposition rate was limited to 2–3 min mL^−1^. The inks were prepared by mixing 1 mg of catalyst in 1 mL of ultra-pure H_2_O (MiliQ, 18 MΩ/cm) and the desired amount of Nafion® D521 solution (5 wt.% in lower aliphatic alcohols and water) to achieve an ionomer content of 25 and 30 wt.% in the dry anode and cathode layer, respectively. The mixture was sonicated for at least 1 h until the catalyst was well dispersed. 1 mL of isopropanol (IPA, ACS reagent, ≥99.5%) was added and the mixture was sonicated for 10 min to reach the adequate dispersion and homogeneity of the ink. This process was scaled up to the desired volume of ink. Subsequent to spraying and drying, the CCM was hot pressed at 5 MPa and 125 °C. The resulting loading of Ir at the anode was 0.4 mg cm^−2^ and the loading of Pt at the cathode was 0.4 mg cm^−2^.

Ca and Sr will leach out in the process of OER electrolysis and could affect strongly the PEMWE performance, likely due to Sr and Ca cations displacing protons in the ionomer and PEM. Therefore, as it is always the case when testing any pristine CCM for PEMWE with a newly developed OER catalyst, the CCM goes through a protocol of activation, which involves an extensive chemical cleaning in diluted H_2_SO_4_ and electrochemical cycling, until the performance of the PEMWE does not change anymore. At this point the Sr and Ca on the surface of catalyst have been completely leached out posing no danger in affecting the PEMWE performance, as we later demonstrate in the durability test.

The CCMs were tested in two different PEMWE setups. The first one is optimized for screening cell components, recording polarization curves, and measuring electrochemical impedance spectroscopy (EIS). Water is fed passively, via natural convection. This ensures stable, steady conditions which are especially important for EIS. The second PEMWE setup is optimized for long-term measurements and the anode and cathode water flow rate was 2.5 L/h. On both the anode and cathode side, a Ti porous sintered layer (PSL) on Ti mesh (PSL/mesh-PTL) compound PTL produced by diffusion bonding^56^ coated with Pt^57^ was deployed. On the cathode, a carbon paper sheet (Spectracarb 2050A-1050) was used as an additional layer contacting the cathode catalyst layer on one, and the PTL on the other side. On both the anode and cathode side Ti-BPPs were employed. The cell active area was 4 cm^2^ and in both setups and tests were carried out at 80 °C and ambient pressure. The polarization curves were measured galvanostatically according to the JRC EU-harmonized procedure^58^, employing a dwell and consecutive recording period of 10 s for each current step. The high-frequency resistance (HFR) was obtained from the high-frequency intercept of the Nyquist plot with the real axis and Tafel slopes are set on from a fit of the linear region between 10–100 mA cm^−2^.

### Reporting summary

Further information on research design is available in the Nature Portfolio Reporting Summary linked to this article.

## Supplementary information

Supplementary Information

Reporting Summary

## Data Availability

The data that support the findings of this study are available within the article and its Supplementary Information files. All other relevant data supporting the findings of this study are available from the corresponding authors upon request.
