# Supplementary material for: Highly active and stable OER electrocatalysts derived from Sr2MIrO6 for proton exchange membrane water electrolyzers
Source: Nat Commun. 2022 Dec 24;13:7935. doi: 10.1038/s41467-022-35631-5 (PMC9789951; doi:10.1038/s41467-022-35631-5)
Supplement: Supplementary file 1 — Supplementary Information [file 41467_2022_35631_MOESM1_ESM.pdf]

Supporting Information

Highly Active and Stable OER Electrocatalysts Derived from Sr_2MIrO_6 for Proton Exchange Membrane Water Electrolyzers

María Retuerto[1,*], Laura Pascual[2], Jorge Torrero[3], Mohamed Abdel Salam[4], Álvaro Tolosana-Moranchel,[1] Diego Gianolio[5], Pilar Ferrer[5], Paula Kayser[6], Vincent Wilke[3], Svenja Stiber[3], Verónica Celorrio[5], Mohamed Mokthar[4], Daniel García Sanchez[3], Aldo Saul Gago[3], Kaspar Andreas Friedrich[3], Miguel Antonio Peña[1], José Antonio Alonso[6], Sergio Rojas[1,*]

[1]Grupo de Energía y Química Sostenibles, Instituto de Catálisis y Petroleoquímica, CSIC. C/Marie Curie 2, 28049, Madrid, Spain

[2]Instituto de Catálisis y Petroleoquímica, CSIC. C/Marie Curie 2, 28049, Madrid, Spain

[3]Institute of Engineering Thermodynamics/Electrochemical Energy Technology, German Aerospace Center (DLR), Pfaffenwaldring 38-40, 70569 Stuttgart, Germany

[4]Chemistry Department, Faculty of Science, King Abdulaziz University, P. O Box 80200, Jeddah, 21589, Saudi Arabia.

[5]Diamond Light Source, Harwell Science and Innovation Campus, Didcot, OX11 0DE, UK

[6]Instituto de Ciencia de Materiales de Madrid, CSIC. C/Sor Juana Inés de la Cruz 3, 28049 Madrid, Spain.

Table of Contents

S1. Comparison with State-of-the-Art Ir catalysts for the OER in acid media.

S2. Crystal structures: Rietveld refinements.

S3. Particle size and crystalline domain sizes.

S4. Calculation of the surface area.

S4.1. Particle size distribution.

S4.2. Electrochemical Active Surface Area.

S5. XPS Ir 4*f* core level region of the initial catalysts.

S6. Electrochemical measurements:

S6.1. Catalyst loading effect.

S6.2. CVs at different scan rates.

S6.3. Specific activities.

S6.4. Durability tests.

S6.5. Turnover Frequency (TOF).

S7. TEM: Immersion of Sr_2CaIrO_6 in the electrolyte.

S8. ICP-OER analysis of the electrolyte after immersion in the electrolyte and after OER cycles.

S9. Surface concentration and Ir 4*f* and O 1*s* core level regions of the catalysts during OER.

S10. XRD results of Sr_2CaIrO_6 along the OER cycling.

S11. *In situ* XAS study of Sr_2CaIrO_6 before and after immersion in the electrolyte.

S12. TEM Study of Sr_2MIrO_6 along the OER cycling.

S12.1. TEM of Sr_2MgIrO_6 and Sr_2ZnIrO_6 after 100 cycles.

S12.2. TEM study of Sr_2CaIrO_6 after 5000 OER cycles.

S13. Comparison of the PEMWE performance of Sr_2CaIrO_6 and commercial Ir_{black} using CCMs with 0.4 mg_{Ir} cm^{-2}_{MEA}.

References

S1. Comparison with State-of-the-Art Ir catalysts for the OER in acid media.

Table S1. State-of-the-art Ir mixed oxide and non-mixed oxide catalysts for the OER in acid electrolyte.

	**E-iR / V @ 10 mAcm^{-2}**	**Tafel slope / mV dec^{-1}**	**i_M Ag^{-1}$_{Ir}$ @ 1.525 V**
**Sr_2CaIrO_6 (this work)**	1.485	33	900
**Sr_2MgIrO_6 (this work)**	1.505	33	260
**Sr_2ZnIrO_6 (this work)**	1.518	38	139
**$AIrO_3$ (A =Sr, Ba)[1]**	1.58	50	13
**Sr_2FeIrO_6 [2,3]**	1.65	90	5.6
**$Sr_2Fe_{0.5}Ir_{0.5}O_4$ [2]**	1.63	50	
**Sr_2CoIrO_6 [2,3]**	1.535	52	73
**Sr_2ScIrO_6 [3]**	1.56	67	35
**Sr_2NiIrO_6 [3]**	1.525	48	115
**$SrTi_{0.67}Ir_{0.33}O_3$ [4]**	1.477	45-80	820
**6H-$SrIrO_3$ [5]**	1.478	60-120	74.7
**$Pr_2Ir_2O_7$ [6]**	1.52	50	351
**$SrCo_{0.9}Ir_{0.1}O_{3-\delta}$ [7]**	1.55	40-70	
**La_2LiIrO_6 [8]**	1.53	50	33.5
**Ba_2BIrO_6 (B= Ce,Tb,Y,La,Pr,Nd)[9]**	1.60	60-130	-
**$Ni_{0.34}Co_{0.46}Ir_{0.2}O_4$[10]**	1.51	40	-
**Sr_2IrO_4 [11]**	1.516	45	392 @1.55V
**Sr_4IrO_6 [11]**	1.517	50	274 @1.55V
**$SrZrO_3$:$SrIrO_3$ (Zr:Ir 1:2)[12]**	1.47	43	1540 @ 1.53 V
**$SrIrO_3$**			314
**$W_{0.57}Ir_{0.43}O_{3-d}$[13]**	1.6	125	1.46 @ 1.53V
**IrO_2-TiO_2[14]**		42(3)	70(3)
**$SrIr_{0.8}Zn_{0.2}O_3$ [15]**	1.55		110 @ 1.55 V
**Ir NSs [16]**	1.485	40	221.8 @ 1.53 V
**$Cu_{1.11}$Ir NCs [17]**	1.516	43.8	73@ 1.51 V
**Ir NCs [18]**	1.5	40.8	
**IrO_2 [8]**	1.56	50	24.5
**IrO_2 [19]**		43.9	37.0 ± 7.9
**IrOOH NSs [20]**	1.574	58(3)	
**$Ir_{44}Pd_{10}$ [21]**	1.456	53.9	1999 @ 1.48 V
**3R-IrO_2 [22]**	1.418	52	691 @ 1.5 V

S2. Crystal structures: Rietveld refinements.

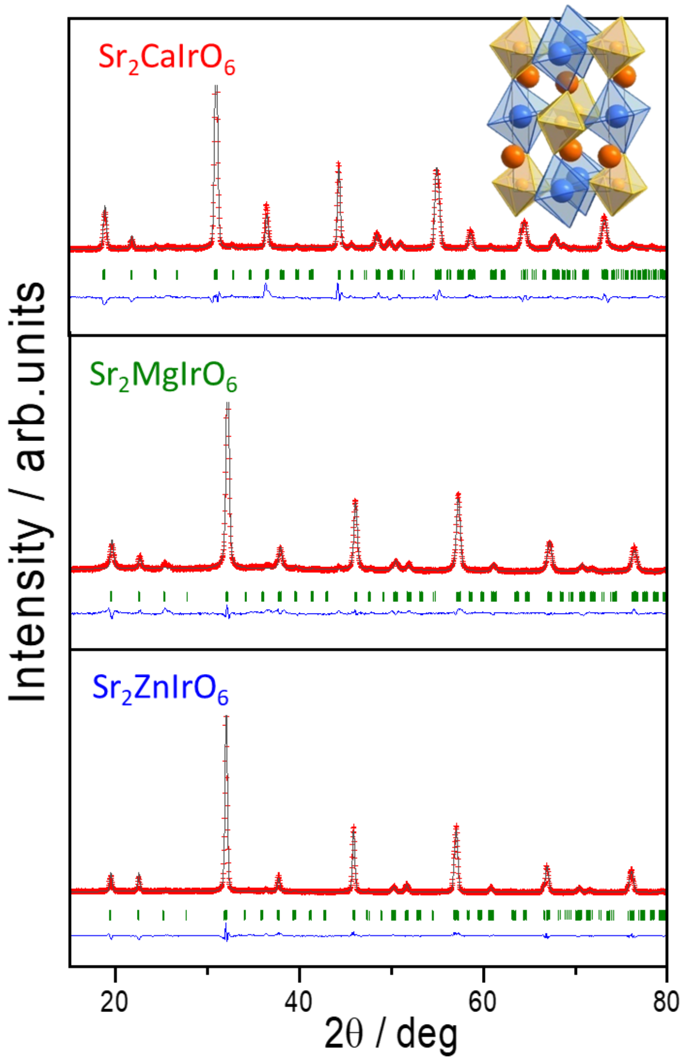

Figure S1. Rietveld refinement of Sr_2MIrO_6 crystal structure using Fullprof program (XRD pattern). Inset: Monoclinic crystal structure involving Ca and Ir 1:1 long-range ordering (Orange octahedra are IrO_6 and blue octahedra are CaO_6, Sr atoms are the red balls).

Table S2. Crystal Structure results from the XRD refinements for Sr_2CaIrO_6, Sr_2ZnIrO_6 and Sr_2MgIrO_6 (space group P$2_1/n$).

	M/Ir Ordering	a / Å	b / Å	c/ Å	Volume cell / Å^3	Monoclinic β angle	<Ir-O>	<M-O>
**Sr_2CaIrO_6**	87(2) %	5.7774(8)	5.7944(9)	8.163(1)	273.26(7)	89.521(4)	1.937(5)	2.248(5)
**Sr_2MgIrO_6**	76(3) %	5.585(2)	5.570(1)	7.863(2)	244.6(1)	89.521(4)	1.955(9)	2.099(9)
**Sr_2ZnIrO_6**	84(3) %	5.6157(3)	5.5821(3)	7.8968(5)	247.54(2)	90.091(5)	1.956(5)	2.038(5)

S3. Particle size and crystalline domain size.

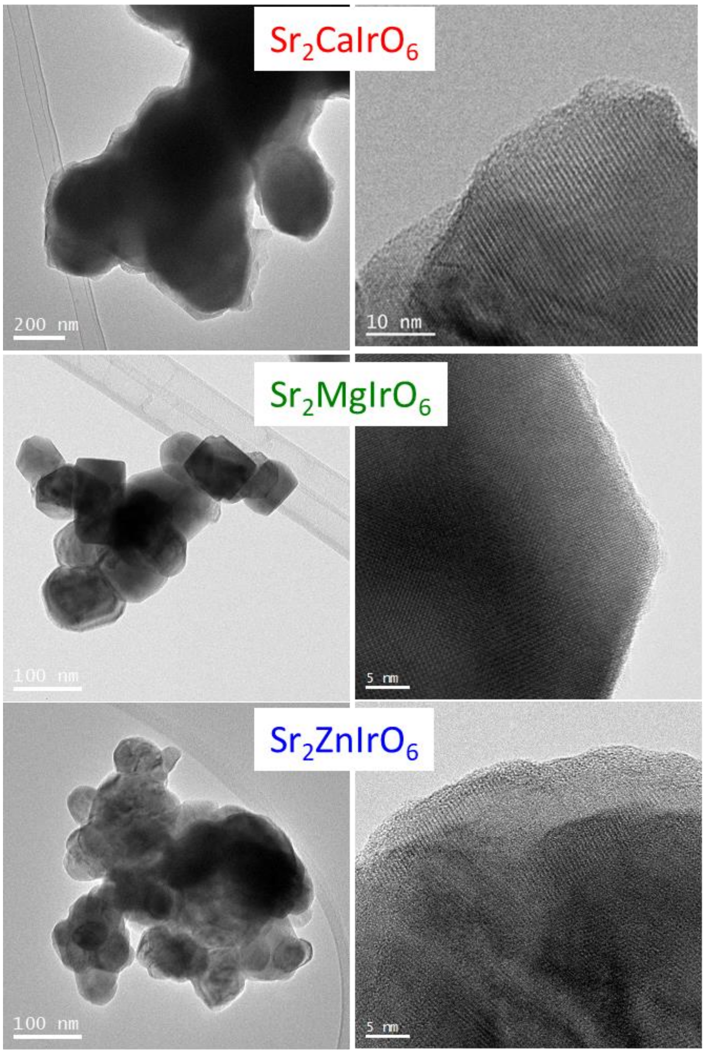

Figure S2. TEM images of Sr_2CaIrO_6, Sr_2MgIrO_6 and Sr_2ZnIrO_6.

S4. Calculation of the surface area.

S4.1. Particle size distribution.

We determine the surface area by the particle size distribution as it has been done in previous studies.[23–25] This method is used when the surface area of the catalysts is too small to be calculated by BET. We assume Sr_2CaIrO_6, Sr_2MgIrO_6 and Sr_2ZnIrO_6 particles as spheres and measure only the ones which are sufficiently isolated that we can represent the diameter *vs*. number of particles.

We determine the mass specific surface area (A_S) using the diameters of the particle sizes (approximating to a spherical geometry) calculated by TEM using the following formula:

$$A_S = {}^{Area}/_{Volumen \times \ Density} = {}^{6}/_{d \times \rho}$$

Where d is the average diameter of the particles calculated by TEM and ρ is the oxides bulk density calculated for Sr_2CaIrO_6, Sr_2MgIrO_6 and Sr_2ZnIrO_6 (P$2_1/n$, Z=2). The values obtained for A_S are shown in Table S3.

Table S3. Particle size, crystalline domain sizes and surface areas of Sr_2CaIrO_6, Sr_2MgIrO_6 and Sr_2ZnIrO_6.

Catalyst	Coherent Domain Size (nm)- from Scherrer Eq. (XRD)*	Grain Size (nm)- from TEM	Mass surface area (A_S) / m^2g^{-1}	ECSA / m^2g^{-1}
**Sr_2CaIrO_6**	35	333	2.1	6
**Sr_2MgIrO_6**	25	62	12.3	20
**Sr_2ZnIrO_6**	30	116	5.6	14

*Note that XRD coherent domain size is much smaller than the grain size, indicating that the grains are composed by several monocrystalline domain regions.

S4.2. Electrochemical Active Surface Area.

The electrochemical active surface areas (ECSA) were calculated form the double-layer capacitance (C_{dl}) of the surface of the oxides. Catalysts inks were prepared by following the procedure detailed in the manuscript but without adding carbon Vulcan. A catalyst loading of 0.25 $mg_{cat}cm^{-2}{}_{geom}$ was used. Cyclic voltammograms in Ar-saturated electrolyte were recorded within a potential window around the open circuit potential so that the electrochemical response would only account to the double-layer charging, without Faradaic processes. The cyclic voltammograms were recorded at different scan rates; 2, 5, 10, 25 and 50 mVs^{-1} (Figure S3). The double-layer charging current (i_c) equals to the product of the scan rate (ν) times C_{dl} at a constant potential. Plotting i_c vs. ν gives a straight line with a slope equal to C_{dl}. For the calculation of the ECSA, C_{dl} is divided by the specific capacitance (C_S) of an atomically flat planar surface of the compounds per unit area under the same electrolyte conditions. A C_S value of 0.035 $mFcm^{-2}$ was used. This value has been previously reported for similar oxides measured in 0.1 M $HClO_4$.[5,26,27] As shown in Table S3, the ECSA values are larger than the A_S values obtained from TEM (or XRD) data. This is because ECSA values are recorded in the electrolyte, where catalyst reconstruction has already occurred (started). By contrary, the A_S values correspond to the fresh catalyst without reconstruction. This observation, supports our claim that catalyst reconstruction during immersion in the electrolyte and OER, results in an open surface.

In addition, the ECSA of Sr_2CaIrO_6 has been measured after OER cycles (Figure S3). In line with the physicochemical results, the ECSA vales of the catalyst after immersion in the electrolyte and after 100 OER cycles are similar, 6 m^2g^{-1} and 7 m^2g^{-1}, respectively. The only appreciable change is the slight decrease of the area after long cycling maybe due to the agglomeration of the particles (see TEM results).

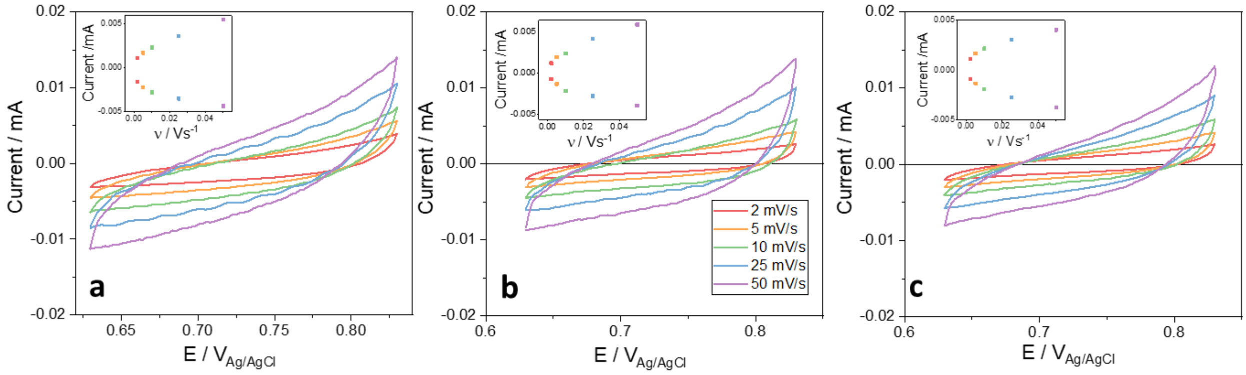

Figure S3. ECSA calculations for Sr_2CaIrO_6 (a) immersed in the electrolyte, (b) after 100 cycles and (c) after 2000 cycles. Upper insets: Plots of i_c *vs.* ν.

S5. XPS Ir 4*f* core level region of the initial catalysts.

The analysis of the Ir 4*f* level in oxides is complex and controversial, and conflicting results are found in the literature. Thus, the binding energies reported for metallic iridium, Ir^{3+}, Ir^{4+} and iridium in higher oxidation states vary strongly depending of the crystalline species involved, and several discrepancies can be found in the literature for the assignation of the peak position to a specific iridium species.

Pfeifer et al.[28,29] reported that, for $4f_{7/2}$ core-level, Ir^{3+} (62.3-65.2 eV) exhibits a reverse binding shift with respect to Ir^{4+} (61.7-61.9 eV). This "special" behavior has been also reported by Vos et al. for films of MnO_x supported on films of IrO_x, used as OER electrocatalysts,[30] with symmetric components for both oxidation states. Also, a comprehensive XPS study of Ir, IrO_2 and $IrCl_3$ pure phases by Freakley et al.[31] used this approach. However, in some cases, the asymmetry of the Ir $4f_{7/2}$ peak in IrO_2 is assigned to additional final states due high degree of core hole screening (core hole - valence electron interaction), as in the work by Kahk et al.[32], using XPS results and Density-Functional Theory. Screening effects are also indicated by Jaramillo et al. on $IrO_x/SrIrO_3$ catalyst for the OER[33] where the $4f_{7/2}$ level for Ir^{4+} appears at 62.3-62.6 eV (a shift of almost 1 eV with respect to bulk IrO_2), and satellite due to screening at 63.4-63.8 eV.

In the last example, the Ir phases are not part of bulk IrO_2, but they are part of a complex system including dispersed iridium oxide on a perovskite oxide. In these cases, when

perovskites, pyrochlores and related phases, and especially when dispersed and/or not pure phases are considered, the approach by Pfeifer et al.[28,29] is not so clear. The bibliographic data about the analysis of the Ir 4*f* level in oxides is complex and controversial for these phases. The BE of the Ir $4f_{7/2}$ level, for metallic iridium, Ir^{3+}, Ir^{4+} and iridium in higher oxidation states, has a great variation depending of the different crystalline species involved, and several discrepancies can be found in the literature for the assignation of the peak position to a specific iridium species.[8,33–39]

Ardizzone et al.[34], for the ternary SnO_2–IrO_2–Ta_2O_5 electrocatalyst, reported Ir^{3+} at 61.7 eV, Ir^{4+} at 62.6 eV, and Ir in oxidation state higher than four at 63.6 eV. Perovskite-like oxides Ca_2IrO_4 and $CaIrO_3$ exhibit components at 62.8 eV, assigned to Ir^{4+}, and 64.1 eV assigned to iridium oxidized beyond +4, see Wu et al.[35] Sadar et al.[36] assigned the peak at 61.7 eV to Ir^{4+}, and the peak at 62.6 eV to iridium oxidized beyond +4, indicating that Ir^{5+} may be present in the pyrochlore $Bi_2Ir_2O_7$ working as OER electrocatalyst. For Iridium-doped strontium titanate ($SrTiO_3$) perovskite as HER electrocatalyst, Nguyen et al.[37] attributed the peaks at 61.4 and 62.7 eV to Ir^{3+} and Ir^{4+} respectively. For similar Ir-doped $SrTiO_3$ photocatalyst, Kawasaki et al.[38] assigned the peak at 62.7 eV to Ir^{4+} and 61.4 eV to Ir^{3+}, indicating that the binding energy of the Ir $4f_{7/2}$ core level has been reported to be between 62.3 and 62.8 eV for Ir^{4+} and between 61.6 and 62.0 eV for Ir^{3+}. Ir^{5+} at 62.8 eV and Ir^{4+}at 62.0 eV have been reported for IrO_x/$BaIrO_3$ used in the OER.[39] Grimaud et at.[8] conclude that the main peak at 64.0 eV for the La_2LiIrO_6 perovskite is due to Ir^{5+}, and that this peak shifts to lower BE (62.9 eV) after OER electrochemical treatments due to the reduction of iridium. Also peaks for Ir^{5+} (63.8-64.0 eV) and Ir^{5+} (62.4) can be identified on the Ba_2PrIrO_6 perovskite.[9] Besides, after the reduction of Ir doped $LaCrO_3$ perovskite[40] and Ir/CeO_2–C nanoparticle electrocatalyst,[41] peaks at 61.6-62.1 eV are assigned to the formation or metallic iridium particles.

From the scattering of results presented above regarding the identification by XPS of the oxidation state of Ir atoms in perovskites, pyrochlores and related phases, as well as for iridium dispersed phases, it can be concluded that the reverse binding shift of Ir^{4+} and Ir^{3+} is not always observed/reported. Both Ir^{0} and Ir^{3+} species display Ir $4f_{7/2}$ peaks in the BE range of 61-62 eV.[34,37,38,40,41] Ir^{4+} peaks at values lower than 62 eV to 63 eV can be also found.[8,33–39] The identification of oxidation states higher than Ir^{4+} begins at values below 63 eV up to higher than 64 eV. [8,9,34–36,39]

Considered the dispersion of the literature data and our XPS results, we have defined a component for the Ir^{4+} contribution in the range 61.6-62.2 eV, for Ir^{4+} in the range 62.2-

63.2 eV, and upper iridium oxides (Ir^{5+}/Ir^{6+}) in the range 63.2-64.4 eV. In the case of the Ir^{3+} component, also a possible contribution of metallic iridium (in the range 61.0-62.1 eV) is possible according to some literature results.[40,41]

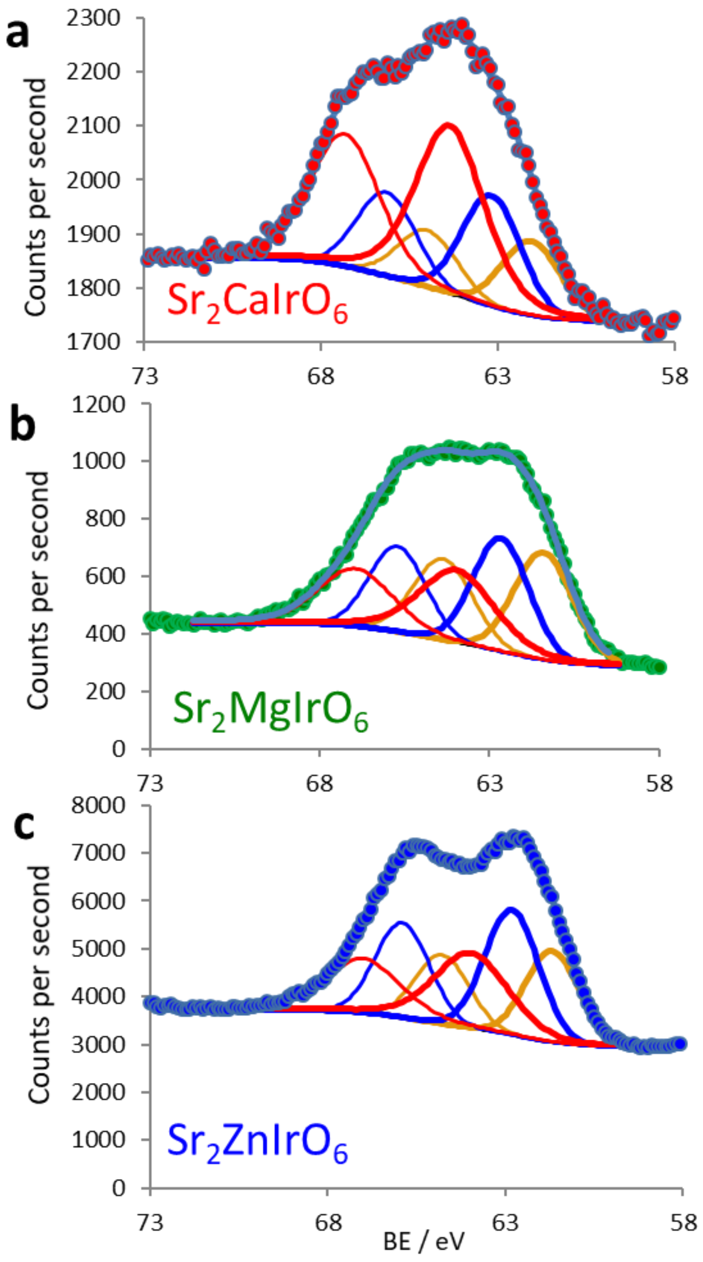

Figure S4. Ir 4*f* core-level region of the initial catalysts. The blue dots are the experimental data, the orange line and the corresponding doublet correspond to Ir^{3+}/Ir^{0} species, the blue line and its corresponding doublet are Ir^{4+}/Ir^{5+} and the red line Ir^{5+}/Ir^{6+}.

S6. Electrochemical measurements.

S6.1. Catalyst loading effect.

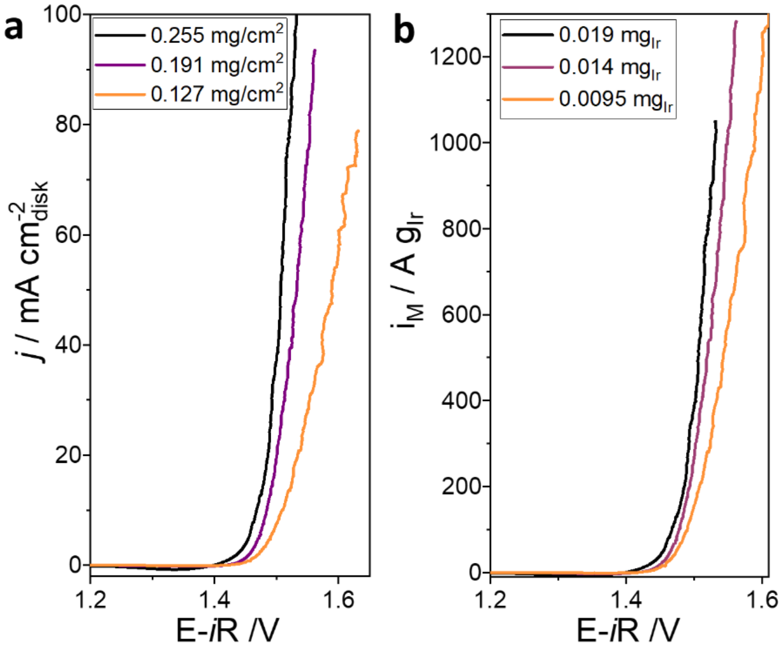

Figure S5. Sr_2CaIrO_6 loading effect (a) current densities normalized to iR, (b) Ir-mass activities normalized to iR.

S6.2. CVs at different scan rates.

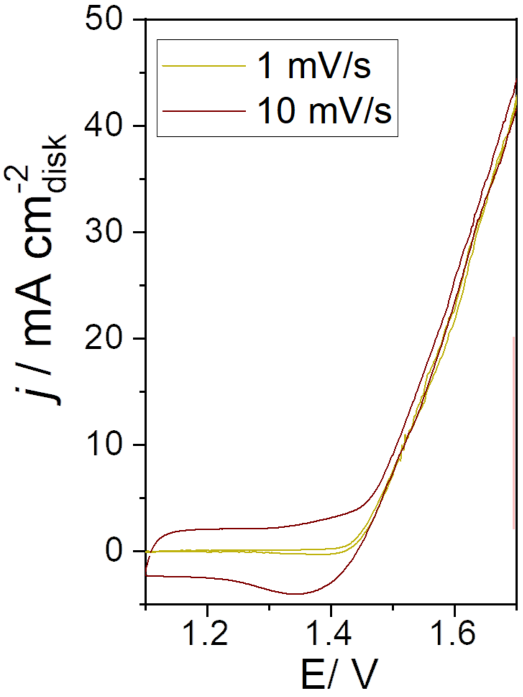

Figure S6. Sr_2CaIrO_6 OER measured at 1mV/s and 10mV/s.

S6.3. Specific activities.

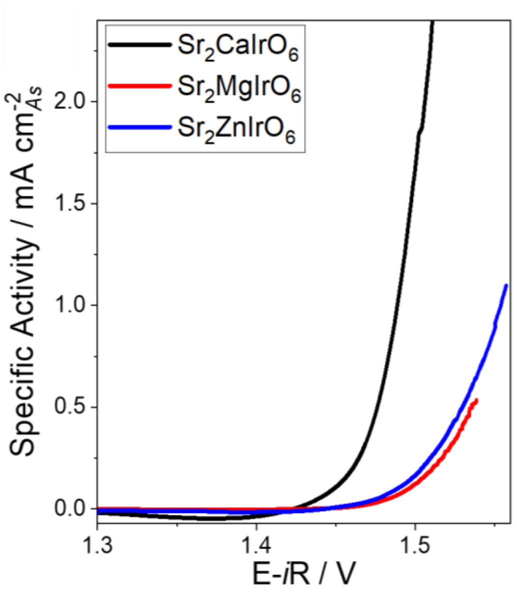

Figure S7. Specific activities for Sr_2MIrO_6 (M= Ca, Mg and Zn) normalized to the mass specific surface area (A_S).

S6.4. Durability Tests.

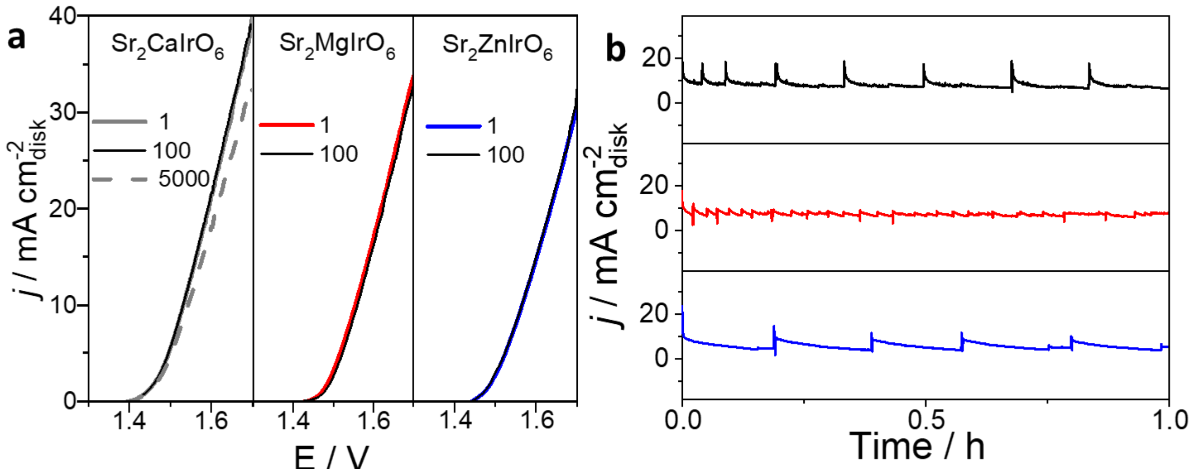

Figure S8. (a) 1st, 100th and 5000th polarization curves recorded with Sr_2CaIrO_6 (red); 1st and 100th curves for Sr_2MgIrO_6 (green); and 1st and 100th OER cycles for Sr_2ZnIrO_6 (blue); measured at 10 mV s^{-1} between 1.2 and 1.7 V *vs.* RHE. (b) Chronoamperometry at 1.53 V for Sr_2CaIrO_6 (red), 1.56 V for Sr_2MgIrO_6 (green), and 1.57 V *vs.* RHE, for Sr_2ZnIrO_6 (blue) at the potentials where j= 10 mAcm^{-2}, during 1 hour.

S6.5. Turnover Frequency (TOF).

Turn over frequency (TOF), i.e., the number of catalytic cycles per active site per time unit is the most representative metric to assess catalytic activity. Accurate TOF values

can only be calculated if the number of active sites is known. Several approaches to determine and report TOF values with iridium (or ruthenium) mixed oxides have been reported. Some authors calculate TOF values by considering that all Ir atoms on the electrode contribute to the catalytic process. However, TOF values obtained from this approach result in a huge underestimation of TOF since only surface atoms are expected to contribute to the catalytic process. Note that depending on the actual geometry of the particles, less than 5 % of total atoms are actually located at the surface of large (> 20 nm) particles.

Ex situ titration methods[42] are on the way to be developed for certain metal oxides, but do not seem to work with iridium oxides. On the other hand, *in situ* techniques such as chemisorption of probe molecules such as CO or NO followed by stripping can be applied with metallic catalysts. Sadly, this approach is not suitable for based in oxides since either the probe molecules do not adsorb on oxidized surfaces or they react once adsorbed.[43]

Electrochemical methods can provide an estimation of the exposed sites. Following previous works, we have calculated the TOF of our catalyst by assuming that the number of accessible Ir surface atoms is represented by the charge between 1.0 and 1.4 V *vs.* RHE.

Since the active phase is formed after reconstruction during OER in acid electrolyte, in order to form the actual catalytic surface, we first recorded 2000 CV cycles between 0.1 and 1.5 V at 100 mV s^{-1} in 0.1 M $HClO_4$. Next, we recorded a CV at 2 mV s^{-1}, see Figure S8, and use this voltammogram to estimate the charge associated to the $Ir^{4+} \rightarrow Ir^{5+}$ redox peak.[44]

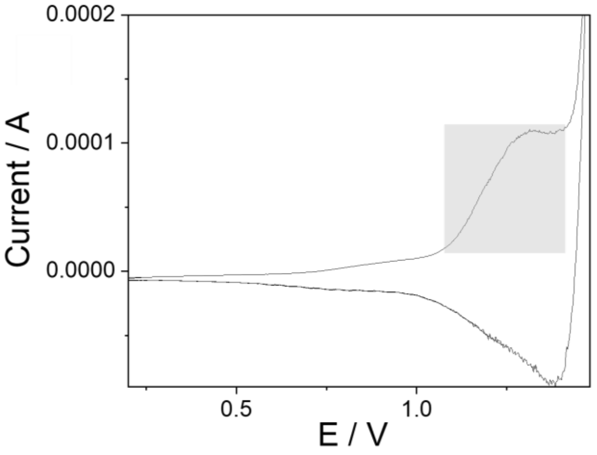

Figure S9. Cyclic voltammogram of Sr_2CaIrO_6 at 2 mV s^{-1} in 0.1 M $HClO_4$ (recorded after 2000 consecutive CVs between 0.1 and 1.5 V at 100 mVs^{-1} in 0.1 M $HClO_4$).

$$TOF = \frac{i_{m,@1.5\,V_{RHE}}[A\ g_{Ir}^{-1}]}{4\ Q_{(1.0-1.4V)}[A\ s\ g_{Ir}^{-1}]}$$

where i_m is the mass activity at a given potential, 391 Ag^{-1}_{Ir} at 1.50 V_{RHE} (*i*R corrected), and Q is the charge associated to the $Ir^{4+} \rightarrow Ir^{5+}$ wave after capacitive current subtraction (138.3 C g^{-1}_{Ir}), which is assumed to be correlated with the number of (surface) Ir active sites. A similar approach has been followed by other authors.[8,19,45,46]

We obtained a TOF (at 1.5V) of 0.71 s^{-1}. For comparison, TOF values reported for several IrO_2 range between 0.05 and 0.6 s^{-1}.[19] This value indicates that the surface Ir (oxide) species formed after reconstruction of Sr_2CaIrO_6 display a high activity, however, higher TOF values have been reported for other catalysts,[7] which record lower mass activities. This feature indicates that the high mass activity of the catalyst obtained from Sr_2CaIrO_6 accounts to both a high intrinsic activity of the surface Ir sites (high TOF) and to a high number of active Ir sites due to the open structure of the catalyst.

Finally, we would lie to remark, again, that comparing TOF values with literature should be taken careful since the values obtained strongly depend on how active surface sites were calculated/determined, so unless the same approach for determining surface site density is used.

S7. TEM: Immersion of Sr_2CaIrO_6 in the electrolyte.

Figure S9 shows the TEM study of Sr_2CaIrO_6-Elec. The SAED analysis of the regions containing Sr, Ca, Ir and O (Figure S6a) show diffraction rings at 4.1 (spot), 3.2 ((110) IrO_2) 2.8 ((200) Sr_2CaIrO_6), 2.2 ((020) IrO_2), 2.0, 1.6 and 1.3 Å, indicating the coexistence of the original perovskite along with IrO_2.

The particles presenting only Ir and O were also analysed (Figure S9b). The SAED patterns show two broad rings, the first one with a maximum at 2.7 Å and the second one at 1.5-1.6 Å that could be ascribed to IrOOH species.

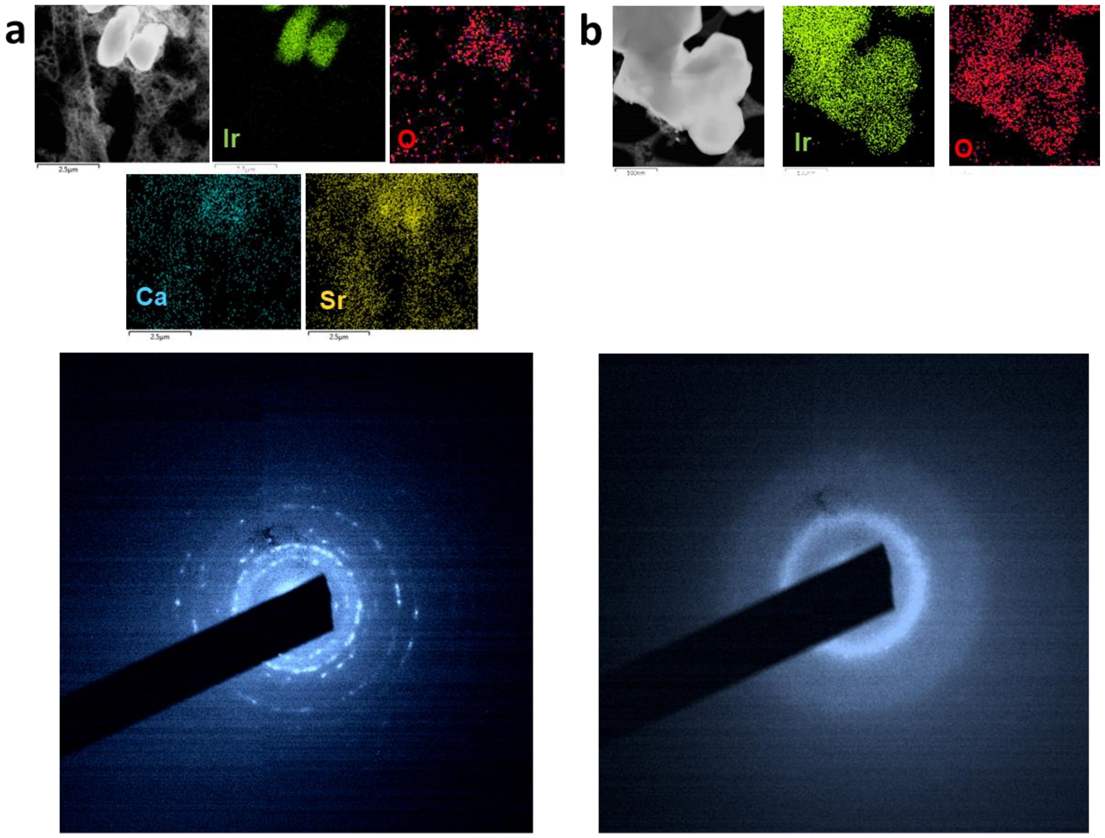

Figure S10. STEM-EDX Mapping and SAED of two different regions of the perovskite immersed in the electrolyte. (a) Fraction of Sr_2CaIrO_6 with Sr, Ca and Ir. (b) Surface regions where only Ir is left.

S8. ICP-OER analysis of the electrolyte after immersion in the electrolyte and after OER cycles.

An Inductively Coupled Plasma Optical Emission Spectrometry (ICP-OES) Analytik Jena PQ 9000 spectrometer was used for the analyses. Specimens for analysis were taken directly from the 0.1 M $HClO_4$ electrolyte. The maximum possible concentration of cations was calculated from the loading of the catalyst and the volume of the electrolyte.

Table S4. ICP-OES chemical analysis of the electrolyte at several stages of the OER reaction with Sr_2CaIrO_6. The maximum expected concentrations for each cation in the electrolyte are 1.74 mg/l for Sr, 0.4 mg/l for Ca and 1.9 mg/l for Ir. Also, the ICP-OES chemical analysis of the electrolyte after immersion of Sr_2ZnIrO_6 are shown where the maximum expected concentrations are 1.66 mg/l for Sr, 0.61 mg/l for Zn and 1.82 mg/l for Ir.

**Sr_2CaIrO_6**	**Cation**	**Conc. / mg l^{-1}**	**SD**	**%**
**Immersed electrolyte**	**Sr**	**0.059**	**0.002**	**3**
	**Ca**	**0.200**	**0.009**	**50**
	**Ir**	**0.023**	**0.004**	**1.2**
**100 cycles**	**Sr**	**1.050**	**0.006**	**60**
	**Ca**	**0.290**	**0.003**	**72**
	**Ir**	**0.023**	**0.001**	**1.2**
**500 cycles**	**Sr**	**1.16**	**0.02**	**67**
	**Ca**	**0.278**	**0.002**	**70**
	**Ir**	**0.021**	**0.004**	**1.1**
**5000 cycles**	**Sr**	**1.04**	**0.01**	**60**
	**Ca**	**0.304**	**0.006**	**76**
	**Ir**	**0.023**	**0.005**	**1.2**
**Sr_2ZnIrO_6**				
**Immersed electrolyte**	**Sr**	**0.05**	**0.0033**	**3.0**
	**Zn**	**0.14**	**0.0010**	**23**
	**Ir**	**0.032**	**0.0014**	**1.8**

S9. Surface concentration and Ir 4*f* and O 1*s* core level regions of the catalysts during OER.

The atomic surface concentration is shown in Figure S5 for Sr_2CaIrO_6 and Sr_2ZnIrO_6. For normalization of the signals, the concentration of every atom was divided by the concentration of carbon, whose intensity is quite constant in every spectrum (in the figures, this normalized value is multiplied by 100).

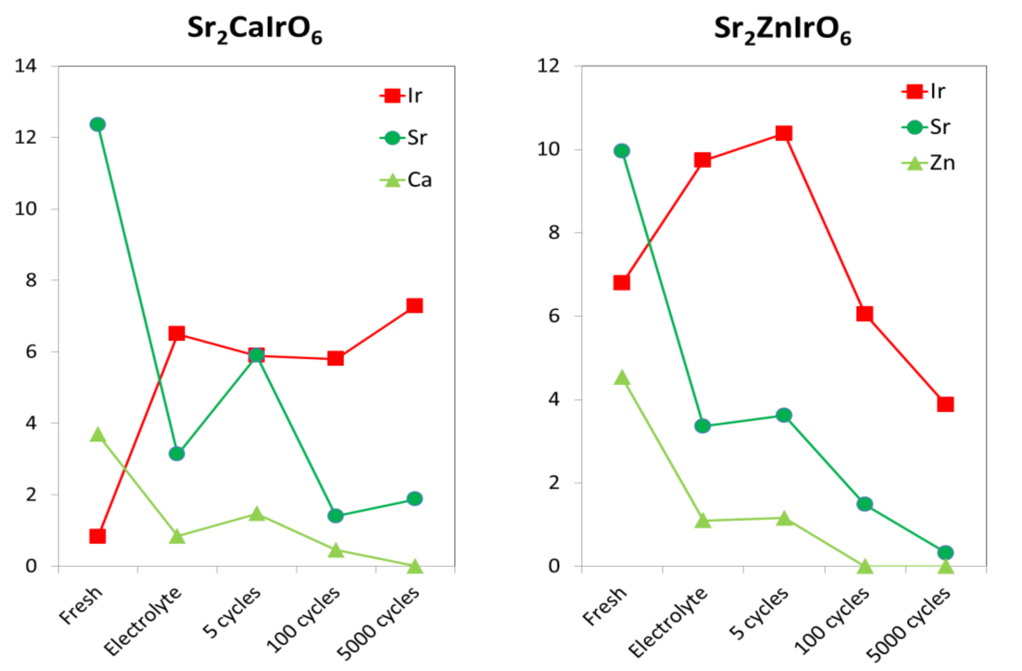

Figure S11. Evolution of the atomic surface concentrations of Ir, Sr and Ca atoms during the OER for Sr_2CaIrO_6 and Sr_2ZnIrO_6 as obtained by XPS.

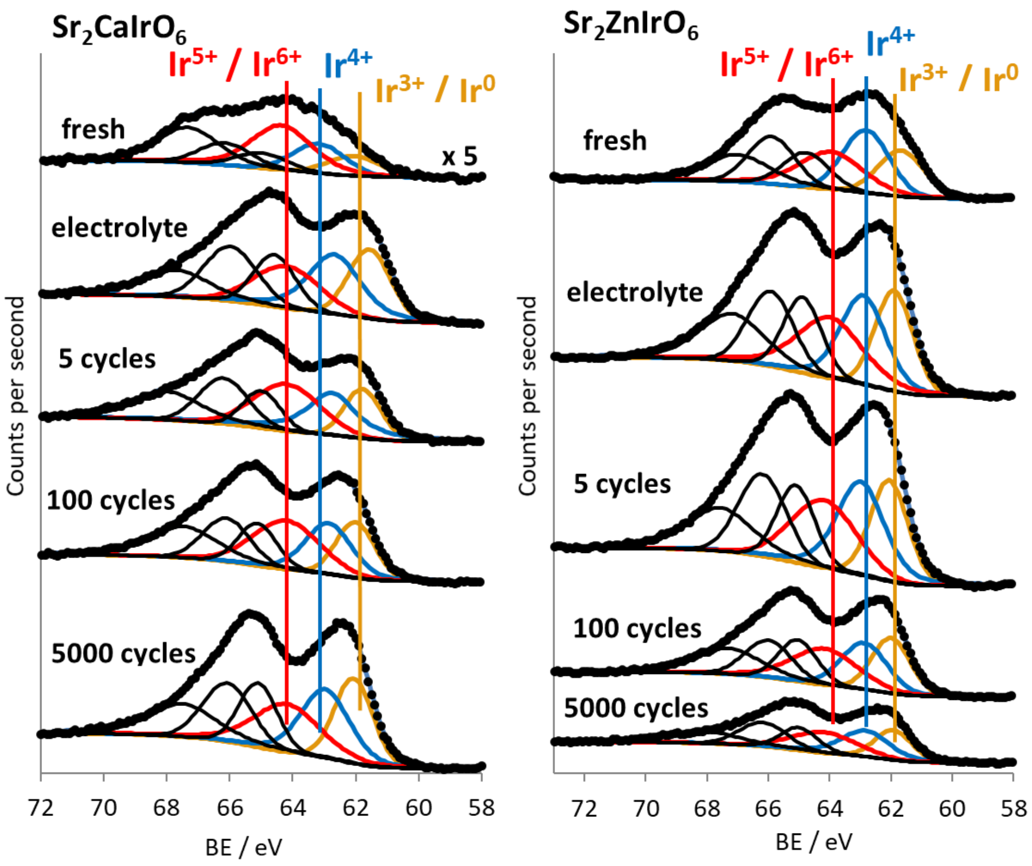

Figure S12. Evolution of the Ir 4*f* core-level region of Sr_2CaIrO_6 and Sr_2ZnIrO_6 catalyst before and after the immersion in the electrolyte (0.1 M HClO4), and after 5, 100 and 5000 OER cycles.

Table S5. Binding energies (eV) for the Ir $4f_{7/2}$ core-levels of the iridium species in the Sr_2MIrO_6 (M=Ca, Zn and Mg) catalysts after the different treatments; immersed in the electrolyte, 5, 100 and 5000 OER cycles.

		**Fresh**	**Electrolyte**	**5 cycles**	**100 cycles**	**5000 cycles**
**Sr_2CaIrO_6**	**Ir^{3+} / Ir^0**	62.0	61.6	61.8	62.0	62.0
	**Ir^{4+}**	63.2	62.7	62.8	62.9	63.0
	**Ir^{5+} / Ir^{6+}**	64.4	64.2	64.2	64.2	64.2
**Sr_2ZnIrO_6**	**Ir^{3+} / Ir^0**	61.7	61.9	62.1	62.0	61.9
	**Ir^{4+}**	62.8	62.9	63.0	62.9	62.8
	**Ir^{5+} / Ir^{6+}**	64.0	64.0	64.2	64.2	64.2
**Sr_2MgIrO_6**	**Ir^{3+} / Ir^0**	61.4				
	**Ir^{4+}**	62.7				
	**Ir^{5+} / Ir^{6+}**	64.0				

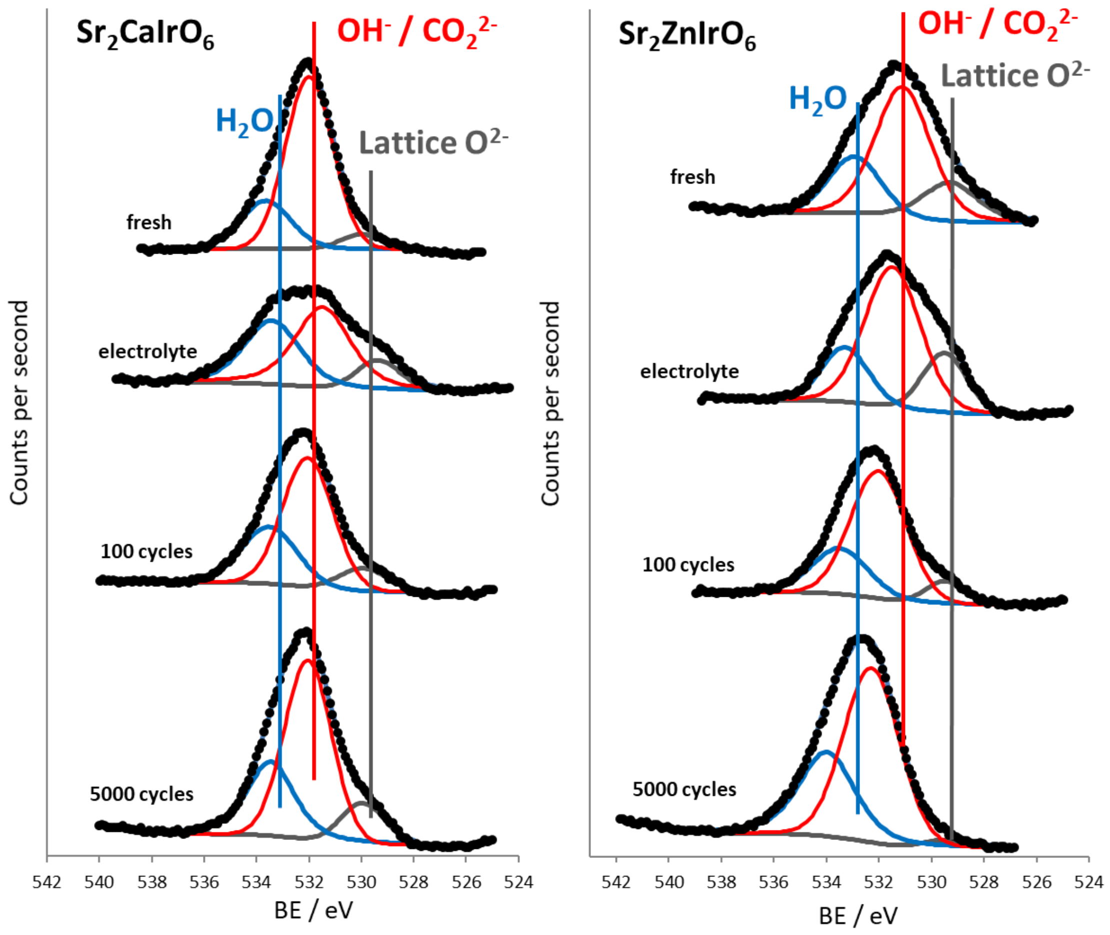

Figure S13. O 1*s* core-level region of the initial catalysts Sr_2CaIrO_6 and Sr_2ZnIrO_6, and of the catalysts recovered after immersion in the electrolyte and after 100 and 5000 OER cycles.

S10. XRD results of Sr_2CaIrO_6 along the OER cycling.

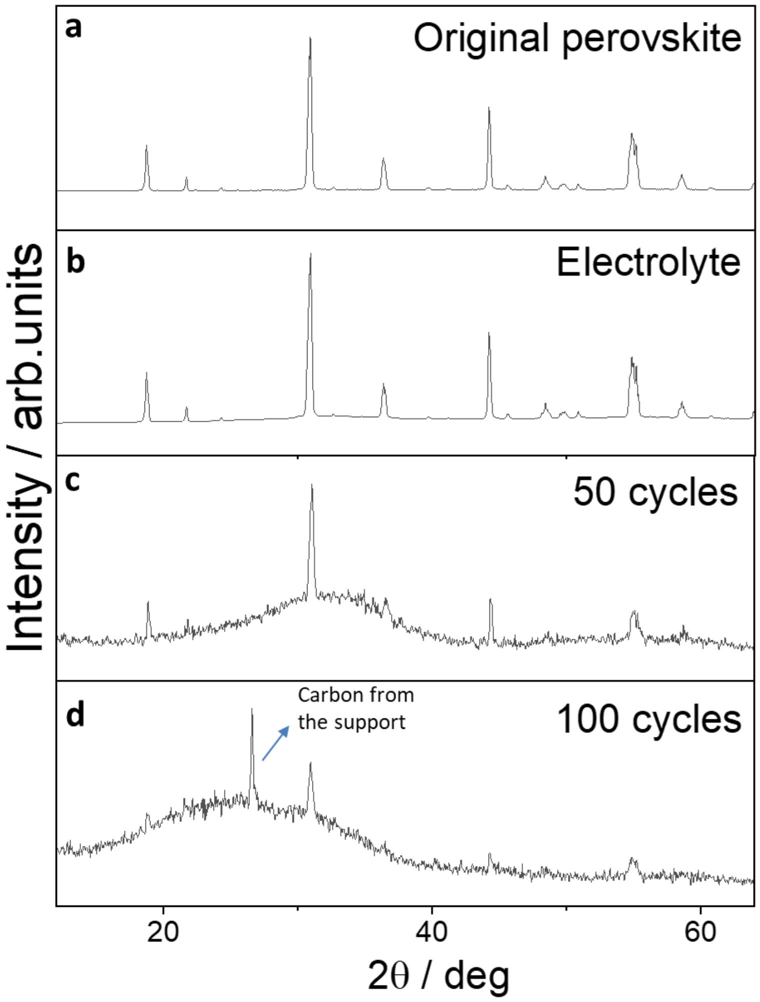

Figure S14. Evolution of the XRD from the initial Sr_2CaIrO_6 catalyst, after immersion in the electrolyte, and after 50 and 100 cycles of OER reaction. The reflection appearing at 26° in the 100 cycles catalyst is due to the carbon from the support.

Table S6. Crystalline domain sizes (nm) from Scherrer Eq. (XRD) of cycled Sr_2CaIrO_6.

**Sr_2CaIrO_6 initial**	**Sr_2CaIrO_6 Electrolyte**	**Sr_2CaIrO_6 50 cycles**	**Sr_2CaIrO_6 100 cycles**
**35**	**33**	**31**	**23**

*Note that XRD coherent domain size is much smaller than the grain size, indicating that the grains are composed by several monocrystalline domain regions.

S11. *In situ* XAS study of Sr_2CaIrO_6 before and after immersion in the electrolyte.

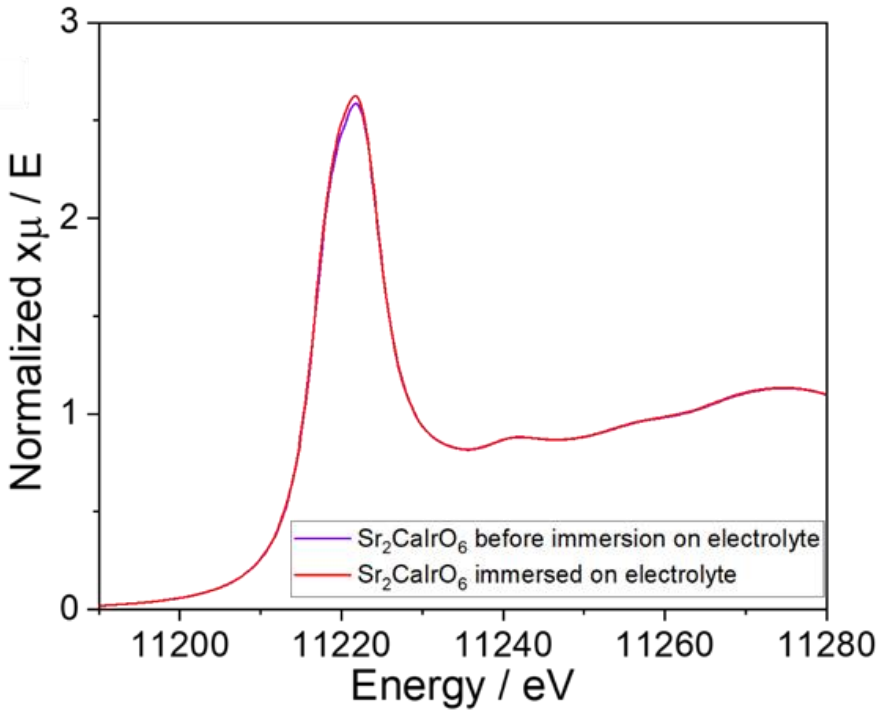

Figure S15. XAS Ir L_3-edge of fresh Sr_2CaIrO_6 catalyst (purple) and after immersion in the electrolyte and at OCP (red).

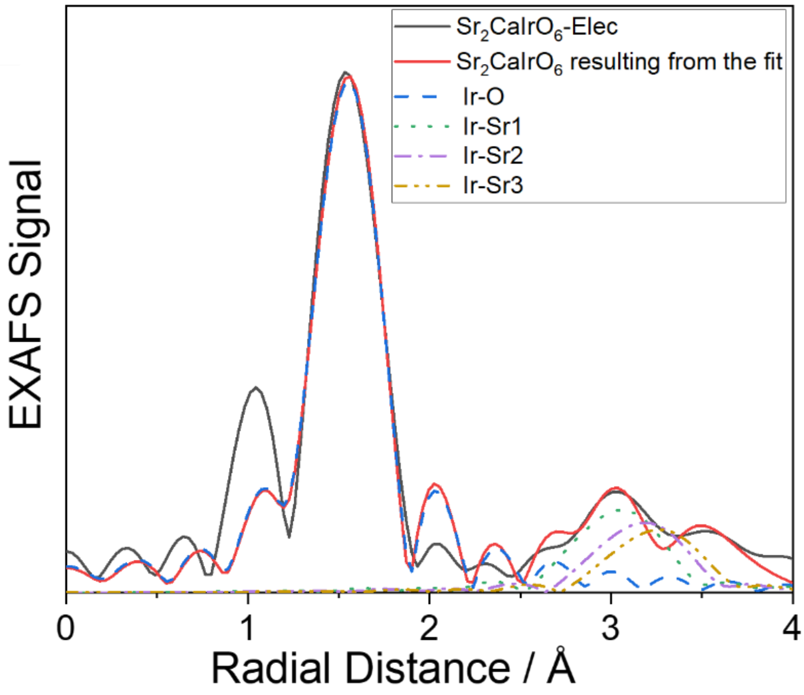

Figure S16. Experimental data and best fit for Sr_2CaIrO_6-Elec, showing major single scattering contributions included in the fit to simulate the EXAFS signal.

Table S7. Best fit results from the structural analysis of Sr_2CaIrO_6 electrode prior to OER cycles at the Ir L_3-edge. N is the coordination number, R is the interatomic distance, σ^2 is the Debye-Waller factor. Same relative energy shift ΔE_0 = 7.8 and S_0^2 = 0.72 were used for all path. R_f is the R-factor, which represents the relative error of the fit and data. Fitting range 3.0 < k <13.1 Å^{-1}, 1.0 < R < 3.4 Å. R_f = 0.025.

**Shell**	**N**	**R / Å**	**σ^2 x 10^3 / Å^2**
**Ir-O**	6	1.92 ± 0.04	1.7 ± 0.7
**Ir-Sr$_1$**	2	3.35 ± 0.07	5.1 ± 1.5
**Ir-Sr$_2$**	2	3.47 ± 0.07	5.5 ± 1.6
**Ir-Sr$_3$**	2	3.55 ± 0.08	5.8 ± 1.8

It is worth noticing that the paths will interfere in the phase space so it would not be correct to assign a peak, observed in the experimental FT-EXAFS data, to a single interatomic bond. Nevertheless, it is possible to observe that the regions where the most relevant changes have been observed during OER cycling correspond to regions where contributions from Ir-Ir paths for IrO_2 and Ir-Ir for IrOOH are expected (Figure S16).

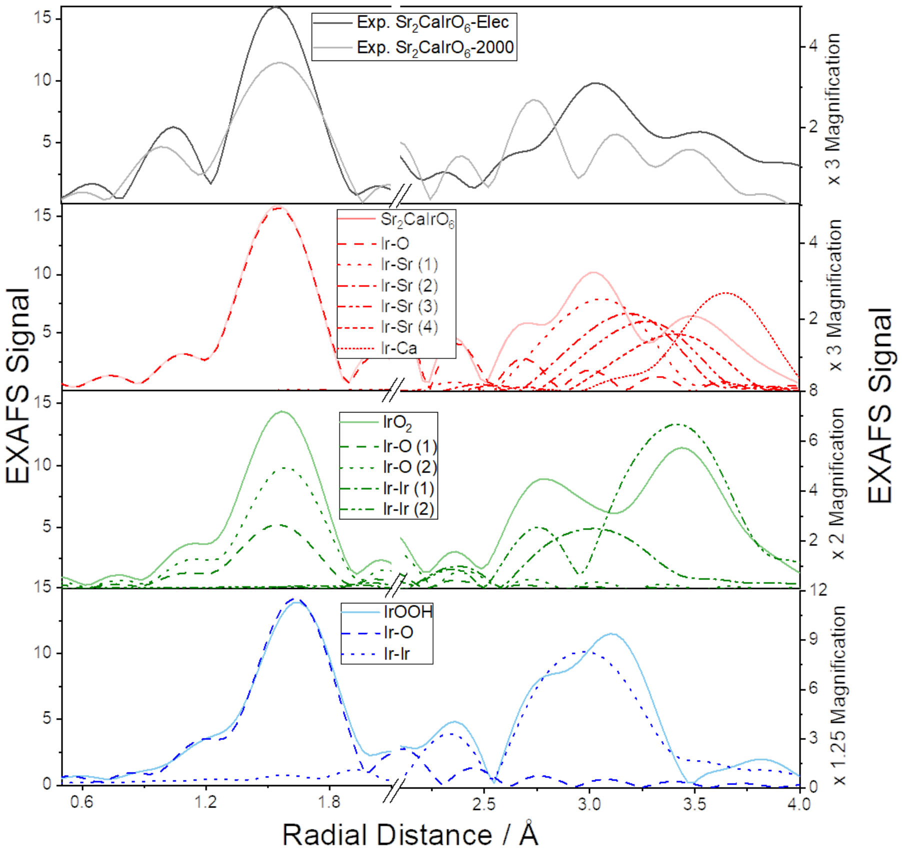

Figure S17. Contributions to FT-EXAFS calculated by using the most relevant single scattering paths for Sr_2CaIrO_6, IrO_2 and IrOOH.

S12. TEM Study of Sr_2MIrO_6 along the OER cycling.

S12.1. TEM of Sr_2MgIrO_6 and Sr_2ZnIrO_6 after 100 cycles.

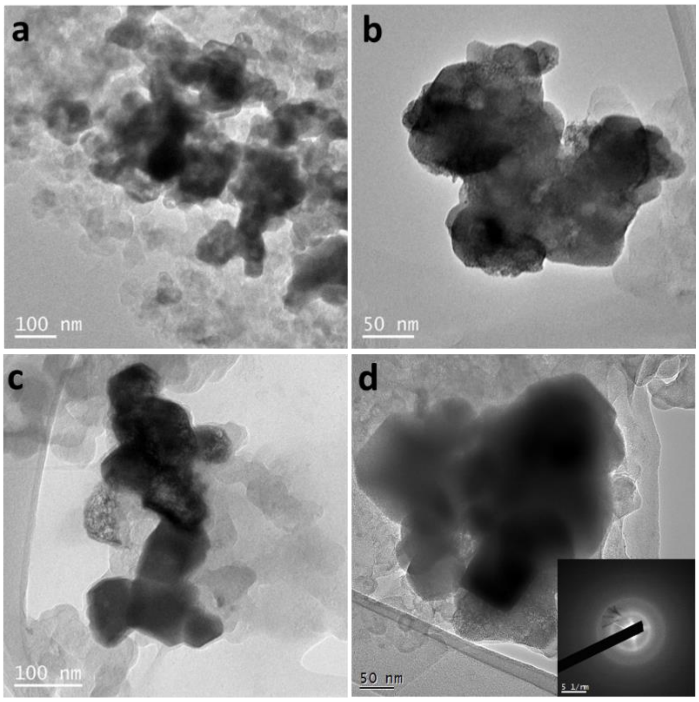

Figure S18. TEM study of (a), (b) Sr_2MgIrO_6 catalyst and (c), (d) Sr_2ZnIrO_6 catalyst after 100 OER cycles.

S12.2. TEM study of Sr_2CaIrO_6 after 5000 OER cycles.

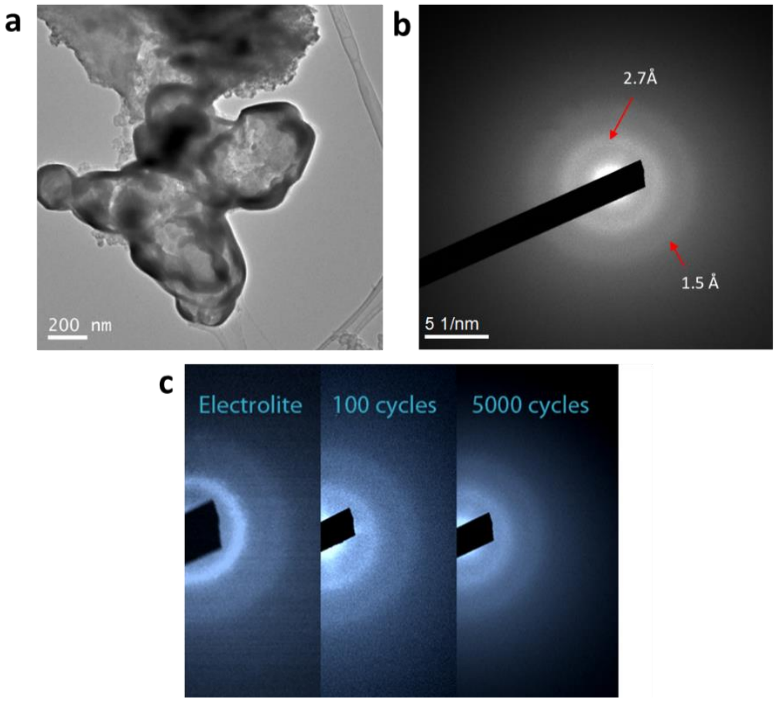

Figure S19. (a) TEM image of a region of Sr_2CaIrO_6 after 5000 OER cycles. (b) SAED of the same region. (c) Evolution of SAED patterns during the OER of the regions containing Ir and O.

S13. Comparison of the PEMWE performance of Sr_2CaIrO_6 and commercial Ir_{black} using CCMs with 0.4 mg_{Ir} cm^{-2}_{MEA}.

The performance of the low Ir loaded CCM produced with Sr_2CaIrO_6 was compared with a CCM produced with a state-of-the-art catalyst (Ir_{black}). A CCM with 0.4 mg_{Ir} cm^{-2} of commercial Ir_{black} (UMICORE) and a N212 membrane was produced by using the coating media recipe reported elsewhere.[47] In order to compare catalytic performances under similar conditions, the commercial Ir_{black} catalyst was deposited onto the CCM without any support, as it was done for Sr_2CaIrO_6. The PEMWE polarization curves obtained under the same conditions (cell components, test station hardware, etc.) along with the high frequency resistance (HFR) and Tafel plots are shown in Figure S20. From the polarization curves in Figure S20a, it can be seen that the performance obtained for the perovskite at a nominal current density of 2 A cm^{-2} is similar than that offered by the benchmark catalyst with the same Ir loading. It should be considered that factors other than the catalyst can also affect the performance of CCMs, for instance the properties of the chosen Ti-PTL or the interface of the catalysts with the PTL. The Ti-PTL used for the measurements reported in our work has a pore size of 52.3 µm,[48] which is quite large compared to the one of felts and sintered plates. Thus, an anode catalyst layer with high in-plane electrical conductivity is required to achieve high cell performances in combination with the Ti-PTL of our work. The HFR values obtained, see Fig. S20b, reveal that the Ti-PTL used in our setup, in combination with the Sr_2CaIrO_6 catalyst layer leads to high cell performances. The enhanced electrocatalytic properties of the electrode are also confirmed by the Tafel slope analysis in Figure S20c. It can be noticed that the Tafel slope of the CCM with Sr_2CaIrO_6 is similar to that offered by the CCM with Ir_{black}, which indicates that the electrode with the perovskite-based catalyst has a kinetics as fast as the electrode with the commercial catalyst.

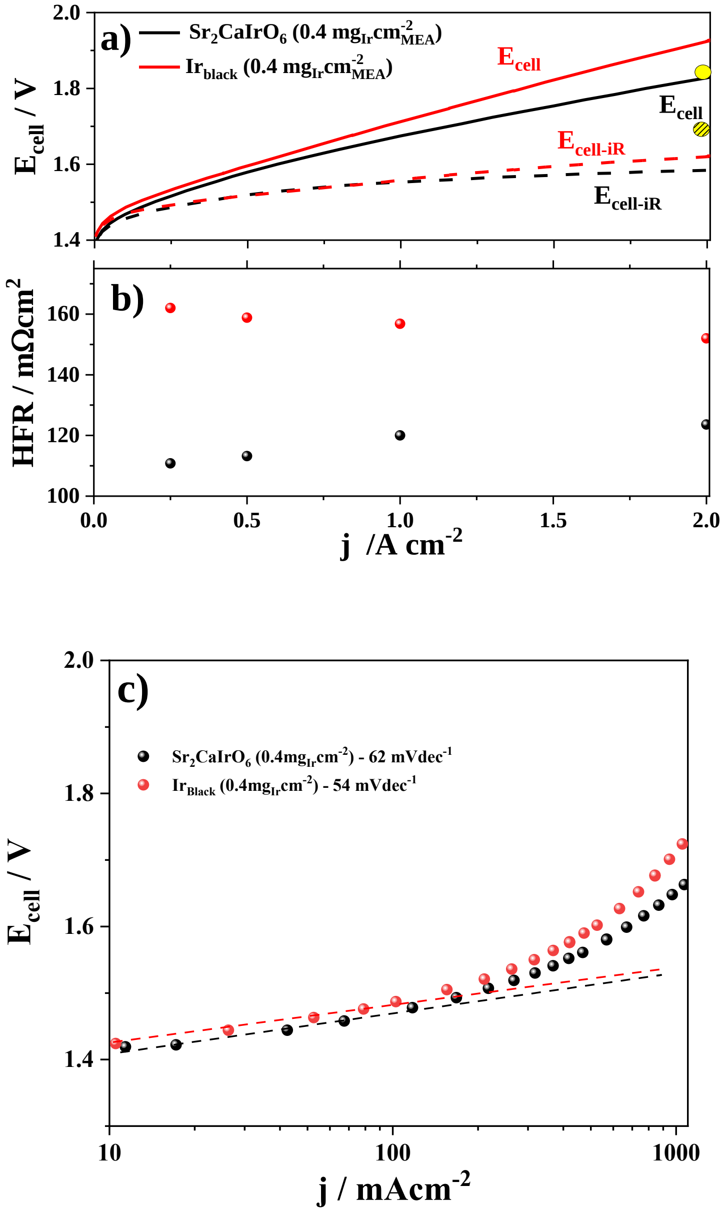

Figure S20. a) Polarization curves for (black) Sr_2CaIrO_6 (0.4 mg_{Ir} cm^{-2}_{MEA}) and (red) commercial Ir_{black} (0.4 mg_{Ir} cm^{-2}_{MEA}) both using Pt/C (commercial) cathode (0.4 mg_{Pt} cm^{-2}_{MEA}). Straight lines represent the measured cell voltage (E_{cell}) and dashed lines are the iR-free cell voltages ($E_{cell\text{-}iR}$ with respect to the current density (j) recorded galvanostatically up to 2 A cm^{-2}. Reference values for E_{cell} ○ and $E_{cell\text{- }iR}$ ▨.[49] b) HFR-values vs. current density obtained by electrochemical impedance spectroscopy. c) Tafel slopes between 10-100 mA cm^{-2} of the *i*R-free cell voltage.

Supplementary References

1. Song, C. W., Suh, H., Bak, J., Bae, H. Bin & Chung, S. Y. Dissolution-Induced Surface Roughening and Oxygen Evolution Electrocatalysis of Alkaline-Earth Iridates in Acid. *Chem* **5**, 3243–3259 (2019).
2. Zhang, R. *et al.* A Dissolution/Precipitation Equilibrium on the Surface of

Iridium-Based Perovskites Controls Their Activity as Oxygen Evolution Reaction Catalysts in Acidic Media. *Angew. Chemie* **131**, 4619–4623 (2019).

3. Retuerto, M. *et al.* How oxidation state and lattice distortion influence the oxygen evolution activity in acid of iridium double perovskites. *J. Mater. Chem. A* **9**, 2980–2990 (2021).
4. Liang, X. *et al.* Activating Inert, Nonprecious Perovskites with Iridium Dopants for Efficient Oxygen Evolution Reaction under Acidic Conditions. *Angew. Chemie Int. Ed.* **58**, 7631–7635 (2019).
5. Yang, L. *et al.* Efficient oxygen evolution electrocatalysis in acid by a perovskite with face-sharing IrO6 octahedral dimers. *Nat. Commun.* **9**, 5236 (2018).
6. Shang, C. *et al.* Electron Correlations Engineer Catalytic Activity of Pyrochlore Iridates for Acidic Water Oxidation. *Adv. Mater.* **31**, 1–6 (2019).
7. Chen, Y. *et al.* Exceptionally active iridium evolved from a pseudo-cubic perovskite for oxygen evolution in acid. *Nat. Commun.* **10**, 572 (2019).
8. Grimaud, A. *et al.* Activation of surface oxygen sites on an iridium-based model catalyst for the oxygen evolution reaction. *Nat. Energy* **2**, 16189 (2017).
9. Diaz-Morales, O. *et al.* Iridium-based double perovskites for efficient water oxidation in acid media. *Nat Commun* **7**, 12363 (2016).
10. Zaman, W. Q. *et al.* Iridium substitution in nickel cobaltite renders high mass specific OER activity and durability in acidic media. *Appl. Catal. B Environ.* **244**, 295–302 (2019).
11. Strickler, A. L., Higgins, D. & Jaramillo, T. F. Crystalline Strontium Iridate Particle Catalysts for Enhanced Oxygen Evolution in Acid. *ACS Appl. Energy Mater.* **2**, 5490–5498 (2019).
12. Liang, X. *et al.* Perovskite-Type Solid Solution Nano-Electrocatalysts Enable Simultaneously Enhanced Activity and Stability for Oxygen Evolution. *Adv. Mater.* **32**, 1–8 (2020).
13. Kumari, S. *et al.* A low-noble-metal W1-xIrxO3-δ water oxidation electrocatalyst for acidic media via rapid plasma synthesis. *Energy Environ. Sci.* **10**, 2432–2440 (2017).
14. Oakton, E. *et al.* IrO2-TiO2: A High-Surface-Area, Active, and Stable Electrocatalyst for the Oxygen Evolution Reaction. *ACS Catal.* **7**, 2346–2352 (2017).
15. Edgington, J., Schweitzer, N., Alayoglu, S. & Seitz, L. C. Constant Change:

Exploring Dynamic Oxygen Evolution Reaction Catalysis and Material Transformations in Strontium Zinc Iridate Perovskite in Acid. *J. Am. Chem. Soc.* **143**, 9961–9971 (2021).

16. Wu, G. *et al.* A general synthesis approach for amorphous noble metal nanosheets. *Nat. Commun.* **10**, 1–8 (2019).
17. Wang, C. *et al.* Synthesis of Cu-Ir nanocages with enhanced electrocatalytic activity for the oxygen evolution reaction. *J. Mater. Chem. A* **3**, 19669–19673 (2015).
18. Pi, Y., Zhang, N., Guo, S., Guo, J. & Huang, X. Ultrathin laminar Ir superstructure as highly efficient oxygen evolution electrocatalyst in broad pH range. *Nano Lett.* **16**, 4424–4430 (2016).
19. Abbott, D. F. *et al.* Iridium Oxide for the Oxygen Evolution Reaction: Correlation between Particle Size, Morphology, and the Surface Hydroxo Layer from Operando XAS. *Chem. Mater.* **28**, 6591–6604 (2016).
20. Weber, D. *et al.* IrOOH nanosheets as acid stable electrocatalysts for the oxygen evolution reaction. *J. Mater. Chem. A* **6**, 21558–21566 (2018).
21. Zhu, J. *et al.* Iridium-Based Cubic Nanocages with 1.1-nm-Thick Walls: A Highly Efficient and Durable Electrocatalyst for Water Oxidation in an Acidic Medium. *Angew. Chemie - Int. Ed.* **58**, 7244–7248 (2019).
22. Fan, Z. *et al.* Extraordinary acidic oxygen evolution on new phase 3R-iridium oxide. *Joule* **5**, 3221–3234 (2021).
23. Suntivich, J., Gasteiger, H. A., Yabuuchi, N. & Shao-Horn, Y. Electrocatalytic Measurement Methodology of Oxide Catalysts Using a Thin-Film Rotating Disk Electrode. *J. Electrochem. Soc.* **157**, B1263 (2010).
24. Retuerto, M. *et al.* Structural effects of LaNiO3 as electrocatalyst for the oxygen reduction reaction. *Appl. Catal. B Environ.* **203**, 363–371 (2017).
25. Lee, Y. *et al.* Synthesis and Activities of Rutile IrO2 and RuO2 Nanoparticles for Oxygen Evolution in Acid and Alkaline Solutions. *J Phys Chem Lett* **3**, 399–404 (2012).
26. McCrory, C. C. L. L., Jung, S., Peters, J. C. & Jaramillo, T. F. Benchmarking Heterogeneous Electrocatalysts for the Oxygen Evolution Reaction. *J. Am. Chem. Soc.* **135**, 16977–16987 (2013).
27. Lin, Y. *et al.* Chromium-ruthenium oxide solid solution electrocatalyst for highly efficient oxygen evolution reaction in acidic media. *Nat. Commun.* **10**, (2019).

28. Pfeifer, V. *et al.* The electronic structure of iridium and its oxides. *Surf. Interface Anal.* **48**, 261–273 (2016).
29. Pfeifer, V. *et al.* The electronic structure of iridium oxide electrodes active in water splitting. *Phys. Chem. Chem. Phys.* **18**, 2292–6 (2016).
30. Vos, J. G., Wezendonk, T. A., Jeremiasse, A. W. & Koper, M. T. M. MnOx/IrOx as Selective Oxygen Evolution Electrocatalyst in Acidic Chloride Solution. *J. Am. Chem. Soc.* **140**, 10270–10281 (2018).
31. Freakley, S. J., Ruiz-Esquius, J. & Morgan, D. J. The X-ray photoelectron spectra of Ir, IrO2 and IrCl3 revisited. *Surf. Interface Anal.* **49**, 794–799 (2017).
32. Kahk, J. M. *et al.* Understanding the Electronic Structure of IrO2 Using Hard-X-ray Photoelectron Spectroscopy and Density-Functional Theory. *Phys. Rev. Lett.* **112**, 117601 (2014).
33. Seitz, L. C. *et al.* A highly active and stable IrOx/SrIrO3 catalyst for the oxygen evolution reaction. *Science* **353**, 1011–1014 (2016).
34. Ardizzone, S. *et al.* Composite ternary SnO2-IrO2-Ta2O5 oxide electrocatalysts. *J. Electroanal. Chem.* **589**, 160–166 (2006).
35. Wu, Y. *et al.* Highly Efficient Oxygen Evolution Activity of Ca2IrO4 in an Acidic Environment due to Its Crystal Configuration. *ACS Omega* **3**, 2902–2908 (2018).
36. Sardar, K. *et al.* Bismuth iridium oxide oxygen evolution catalyst from hydrothermal synthesis. *Chem. Mater.* **24**, 4192–4200 (2012).
37. Nguyen, C. Van, Trang, T. N. Q., Pham, H. Q., Thu, V. T. H. & Ho, V. T. T. One-step heating hydrothermal of iridium-doped cubic perovskite strontium titanate towards hydrogen evolution. *Mater. Lett.* **282**, 128686 (2021).
38. Kawasaki, S. *et al.* Electronic structure and photoelectrochemical properties of an Ir-doped SrTiO3 photocatalyst. *J. Phys. Chem. C* **118**, 20222–20228 (2014).
39. Li, N. *et al.* Identification of the Active-Layer Structures for Acidic Oxygen Evolution from 9R-BaIrO 3 Electrocatalyst with Enhanced Iridium Mass Activity. *J. Am. Chem. Soc.* **143**, 18001–18009 (2021).
40. Coşkun, M. *et al.* Frequency and temperature dependent electrical and dielectric properties of LaCrO3 and Ir doped LaCrO3 perovskite compounds. *J. Alloys Compd.* **740**, 1012–1023 (2018).
41. Qin, B. *et al.* A novel Ir/CeO2-C nanoparticle electrocatalyst for the hydrogen oxidation reaction of alkaline anion exchange membrane fuel cells. *RSC Adv.* **7**,

31574–31581 (2017).

42. Wachs, I. E. Number of surface sites and turnover frequencies for oxide catalysts. *J. Catal.* **405**, 462–472 (2022).
43. Alia, S. M., Hurst, K. E., Kocha, S. S. & Pivovar, B. S. Mercury Underpotential Deposition to Determine Iridium and Iridium Oxide Electrochemical Surface Areas. *J. Electrochem. Soc.* **163**, F3051–F3056 (2016).
44. Geiger, S. *et al.* The stability number as a metric for electrocatalyst stability benchmarking. *Nat. Catal.* **1**, 508–515 (2018).
45. Wang, L. *et al.* Nanostructured Ir-supported on Ti4O7 as a cost-effective anode for proton exchange membrane (PEM) electrolyzers. *Phys. Chem. Chem. Phys.* **18**, 4487–4495 (2016).
46. Nong, H. N., Gan, L., Willinger, E., Teschner, D. & Strasser, P. IrOx core-shell nanocatalysts for cost- and energy-efficient electrochemical water splitting. *Chem. Sci.* **5**, 2955–2963 (2014).
47. Bernt, M., Schröter, J., Möckl, M. & Gasteiger, H. A. Analysis of Gas Permeation Phenomena in a PEM Water Electrolyzer Operated at High Pressure and High Current Density. *J. Electrochem. Soc.* **167**, 124502 (2020).
48. Stiber, S. *et al.* Porous Transport Layers for Proton Exchange Membrane Electrolysis Under Extreme Conditions of Current Density, Temperature, and Pressure. *Adv. Energy Mater.* **11**, 2100630 (2021).
49. Bernt, M., Siebel, A. & Gasteiger, H. A. Analysis of Voltage Losses in PEM Water Electrolyzers with Low Platinum Group Metal Loadings. *J. Electrochem. Soc.* **165**, F305–F314 (2018).
